# *Carpesii fructus* extract exhibits neuroprotective effects in cellular and *Caenorhabditis elegans* models of Parkinson's disease

**DOI:** 10.1111/cns.14515

**Published:** 2023-10-31

**Authors:** Feng‐Dan Zhu, Bin‐Ding Wang, Da‐Lian Qin, Xiao‐Hui Su, Lu Yu, Jian‐Ming Wu, Betty Yuen‐Kwan Law, Min‐Song Guo, Chong‐Lin Yu, Xiao‐Gang Zhou, An‐Guo Wu

**Affiliations:** ^1^ Sichuan Key Medical Laboratory of New Drug Discovery and Drugability Evaluation, Luzhou Key Laboratory of Activity Screening and Druggability Evaluation for Chinese Materia Medica, Key Laboratory of Medical Electrophysiology of Ministry of Education School of Pharmacy, Southwest Medical University Luzhou China; ^2^ Institute of Chinese Materia Medica, China Academy of Chinese Medical Sciences Beijing China; ^3^ State Key Laboratory of Quality Research in Chinese Medicine, Macau University of Science and Technology Taipa China

**Keywords:** 6‐OHDA, *Caenorhabditis elegans*, *Carpesii fructus* extract, MAPK pathway, Parkinson's disease, PC‐12 cells, α‐Synuclein

## Abstract

**Objective:**

Parkinson's disease (PD) is a debilitating neurodegenerative disorder characterized by the progressive loss of dopaminergic neurons in the substantia nigra. Despite extensive research, no definitive cure or effective disease‐modifying treatment for PD exists to date. Therefore, the identification of novel therapeutic agents with neuroprotective properties is of utmost importance. Here, we aimed to investigate the potential neuroprotective effects of *Carpesii fructus* extract (CFE) in both cellular and *Caenorhabditis elegans* (*C. elegans*) models of PD.

**Methods:**

The neuroprotective effect of CFE in H_2_O_2_‐ or 6‐OHDA‐induced PC‐12 cells and α‐synuclein‐overexpressing PC‐12 cells were investigated by determining the cell viability, mitochondrial damage, reactive oxygen species (ROS) production, apoptosis, and α‐synuclein expression. In NL5901, BZ555, and N2 worms, the expression of α‐synuclein, motive ability, the viability of dopaminergic neurons, lifespan, and aging‐related phenotypes were investigated. The signaling pathway was detected by Western blotting and validated by employing small inhibitors and RNAi bacteria.

**Results:**

In cellular models of PD, CFE significantly attenuated H_2_O_2_‐ or 6‐OHDA‐induced toxicity, as evidenced by increased cell viability and reduced apoptosis rate. In addition, CFE treatment suppressed ROS generation and restored mitochondrial membrane potential, highlighting its potential as a mitochondrial protective agent. Furthermore, CFE reduced the expression of α‐synuclein in wide type (WT)‐, A53T‐, A30P‐, or E46K‐α‐synuclein‐overexpressing PC‐12 cells. Our further findings reveal that CFE administration reduced α‐synuclein expression and improved its induced locomotor deficits in NL5901 worms, protected dopaminergic neurons against 6‐OHDA‐induced degeneration in BZ555 worms, extended lifespan, delayed aging‐related phenotypes, and enhanced the ability of stress resistance in N2 worms. Mechanistic studies suggest that the neuroprotective effects of CFE may involve the modulation of the MAPK signaling pathway, including ERK, JNK, and p38, whereas the interference of these pathways attenuated the neuroprotective effect of CFE in vitro and in vivo.

**Conclusion:**

Overall, our study highlights the potential therapeutic value of CFE as a neuroprotective agent in the context of PD. Furthermore, elucidation of the active compounds of CFE will provide valuable insights for the development of novel therapeutic strategies for PD.

## Graphical abstract

*Carpesii fructus extract* (CFE) attenuates H_2_O_2_‐ or 6‐OHDA‐induced toxicity and reduce α‐synuclein to inhibit ROS production and restore MMP in PC‐12 cells and *Caenorhabditis elegans*, resulting in the improvement of dopaminergic neurons and motor ability in PD. These effects are involved the regulation of MAPK signaling pathway.

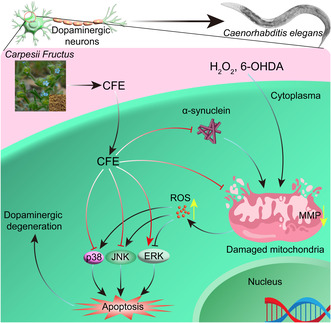

## 1 INTRODUCTION

Parkinson's disease (PD) is a complex neurodegenerative disorder characterized by the progressive loss of dopaminergic neurons in the substantia nigra pars compacta (SNpc) region of the brain.^1^ This loss leads to motor impairments such as tremors, bradykinesia, and postural instability, accompanied by non‐motor symptoms including cognitive decline and psychiatric disturbances.^2^, ^3^, ^4^ Despite extensive research efforts, effective treatments for PD remain elusive, highlighting the need for novel therapeutic strategies.

The pathogenesis of PD revolves around intricate molecular processes, with α‐synuclein aggregation and subsequent formation of Lewy bodies acting as a prominent hallmark.^5^ These aberrant protein accumulations instigate a cascade of events, including mitochondrial dysfunction,^6^ oxidative stress,^6^ impaired protein degradation pathways,^7^ and neuroinflammation.^8^ Furthermore, emerging evidence suggests the involvement of environmental factors, including pesticides, heavy metals, and mitochondrial toxins, in PD pathogenesis, accentuating the intricate interplay between genetic predisposition and external triggers.^9^, ^10^, ^11^ Growing evidence suggests that oxidative stress plays a pivotal role in PD pathogenesis. Specifically, α‐synuclein, when misfolded or aggregated, can induce oxidative stress by disrupting mitochondrial function and increasing the production of reactive oxygen species (ROS).^12^ Moreover, oxidative stress can further exacerbate α‐synuclein aggregation, creating a vicious cycle that accelerates neuronal damage.^13^ The use of sophisticated cellular and animal models has proven instrumental in elucidating the complex mechanisms underlying PD.^14^, ^15^, ^16^, ^17^, ^18^ To date, in vitro and in vivo models overexpressing α‐synuclein or utilizing neurotoxic agents have significantly contributed to our understanding of PD‐related neurodegeneration and the potential for neuroprotective strategies.

Natural products derived from plants have gained considerable attention in recent years due to their potential therapeutic properties against various diseases, including neurodegenerative disorders.^13^, ^19^, ^20^, ^21^ To date, numerous natural products have been reported to have neuroprotective effects in cellular and animal models of PD.^22^ *Carpesii fructus*, a medicinal herb traditionally used in East Asian medicine, has been reported to possess various pharmacological activities, including antioxidant,^23^ antiparasitic,^24^ anti‐inflammatory,^25^ and anti‐tumor properties.^26^ However, the neuroprotective effect of *Carpesii fructus* and its underlying mechanisms of action in PD models have not been extensively investigated. In this study, we aimed to evaluate the neuroprotective effects of *Carpesii fructus* extract (CFE) using both cellular and *Caenorhabditis elegans* (*C. elegans*) models of PD. The results show that CFE attenuated H_2_O_2_‐ or 6‐HDA‐induced toxicity, protected mitochondrial normal function, and reduced α‐synuclein expression in cellular models of PD. Additionally, CFE reduced α‐synuclein expression and improved its induced locomotor deficits, protected dopaminergic neurons against 6‐OHDA‐induced degeneration, extended lifespan, delayed aging‐related phenotypes, and enhanced the ability of stress resistance in *C. elegans*. These effects are mediated by the MAPK signaling pathways. Thus, the findings from this study have significant implications for the development of novel therapeutics for PD and may shed light on the potential neuroprotective mechanisms of CFE.

## 2 MATERIALS AND METHODS

### 2.1 Chemicals, plasmids, and antibodies

SCH772984 was purchased from Topscience Co., Ltd.; N‐acetyl‐L‐cystine (NAC, A7520), Hoechst 33342 (B2261), PI (P4170), and dihydroethidium (DHE, 309800) were purchased from Sigma Chemical Co. Dulbecco's modified Eagle's medium (DMEM), Trypsin–EDTA, and penicillin–streptomycin were purchased from Thermo Fisher Scientific Inc. Fetal bovine serum (FBS, #164210) was obtained from Procell Life Science & Technology Co., Ltd. YFP‐CL1 (11950), EGFP‐α‐synuclein‐WT (40822), EGFP‐α‐synuclein‐A53T (40823), and pHM6‐α‐synuclein‐A53T (40825) plasmids were purchased from Addgene (Addgene Inc.). EGFP‐N1 (6085/1/1), EGFP‐α‐synuclein‐A30P (PPL01823‐2b), and EGFP‐α‐synuclein‐E46K (PPL01823‐2c) plasmids were purchased from Public Protein/Plasmid Library (PPL). The commercially available antibodies used in this study include Bax (14,796S), Bcl‐2 (3498S), ERK (4695S), JNK (4668S), α‐actinin (3134S), and p‐JNK (4668S) from Cell Signaling Technology Inc. (CST); p38 MAPK (sc‐7972), p‐p38 MAPK (sc‐166,182), Caspase‐3 (sc‐390,394), and β‐actin (sc‐47,778) from Santa Cruz Biotechnology Inc; GAPDH (60004‐1‐Ig), Caspase‐9 (10380‐1‐AP), and PARP1 (13371‐I‐AP) from Proteintech Group, Inc; p‐ERK (ap0974) from ABclonal Biotechnology Co., Ltd.; and GFP (MO49‐3) from MBL International. The specificity of all antibodies was verified by the suppliers.

### 2.2 Preparation of *Carpesii fructus* extract

Dried *Carpesii fructus* was purchased from Tong Ren Tang Company and authenticated by Professor Can Tang. A voucher specimen (No. CF‐2021‐001) has been deposited at Southwest Medical University for future reference. *Carpesii fructus* extract (CFE) was prepared using a previously established method.^27^ Briefly, the dried fruit was ground into a fine powder and extracted with 75% ethanol using an ultrasonication‐assisted extraction technique. The extract was then filtered, concentrated under reduced pressure, and lyophilized to obtain a powdered form. The extract was stored at −20°C until further use.

### 2.3 UHPLC‐DAD‐Q/TOF‐MS/MS analysis

The constituents of the CFE were identified using a Shimadzu UHPLC system. This system encompassed an LC‐3 AD solvent dispenser, SIL30ACXR autosampler, CTO‐30 AC column oven, DGU‐20A3 degasser, and CBM‐20A controller. To separate the samples, an Agilent UHPLC Zorbax EcLipse Plus C_18_ column (100 mm × 2.1 mm, 1.8 μm) with a flow rate of 0.3 mL/min was utilized. The column temperature remained constant at 40°C throughout the analysis. The mobile phase consisted of a solution of water with 0.1% formic acid (A) and acetonitrile containing 0.1% formic acid (B). The elution gradient was programmed as follows: From 0 to 15 min, there was a gradual increase from 10% to 95% of solvent B; from 15.01 to 19 min, there was a linear increase from 95% to 10% of solvent B. An AB SCIEX triple TOF X500R instrument equipped with a Duo Spray ion source was employed for mass spectrometry analysis. Negative electrospray ionization (ESI) mode was used, with the following parameters: ion spray voltage set to −4500 V, curtain gas maintained at 35 psi, ion source temperature set at 550°C, declustering potential (DP) set at −100 V, nebulizer gas (GS1) at 55 psi, and heater gas (GS2) at 55 psi. The mass scan range for the MS scan was set from 50 to 1600 Da. For data processing and interpretation, PeakView 1.4 software was employed in the analysis.

### 2.4 Cell culture

PC‐12 cells, a commonly used model for studying neuronal function, were obtained from the American Type Culture Collection (ATCC). Stable GFP‐LC3 U87 cells were provided by Dr. Xiaoming Zhu at Macau University of Science and Technology. Cells were cultured in high‐glucose DMEM or α‐MEM supplemented with 10% fetal bovine serum (FBS) and 1% penicillin–streptomycin (PS). The cells were maintained in a humidified incubator at 37°C with 5% CO_2_.

### 2.5 Detection of intracellular reactive oxygen species (ROS) levels

To evaluate the effect of CFE on ROS levels in PC‐12 cells, a fluorescent probe, dihydroethidium (DHE), was used. After treatment, PC‐12 cells were incubated with DHE (10 μM) for 30 min at 37°C. The cells were then washed with phosphate‐buffered saline (PBS) to remove excess DHE. The intracellular distribution and intensity of DHE fluorescence, reflecting ROS levels, were visualized and analyzed using a Nikon ECLIPSE 80i fluorescence microscope. Representative images were captured from multiple fields, ensuring a sufficient number of cells were analyzed. The fluorescence intensity of DHE in individual cells was measured, and the mean fluorescence intensity (MFI) was calculated. Additionally, the ROS levels were measured by a BD FACSVerse™ flow cytometer (BD Bioscience). Briefly, following the incubation period, the cells were washed with PBS to remove excess DHE. The fluorescence intensity of DHE was measured by the flow cytometer. A sufficient number of events (10,000 cells) were acquired for each sample.

### 2.6 MTT assay

Cell viability was determined using the 3‐(4,5‐dimethylthiazol‐2‐yl)‐2,5‐diphenyltetrazolium bromide (MTT) assay.^28^ After the treatment period, the culture medium was removed, and the cells were incubated with MTT solution (0.5 mg/mL) for 4 h at 37°C. The formazan crystals formed were solubilized by adding dimethyl sulfoxide (DMSO) and gently shaking the plates for 10 min. The absorbance was measured at a wavelength of 570 nm using a microplate reader.

### 2.7 Detection of cell apoptosis

Cell apoptosis was evaluated using flow cytometry analysis with Annexin V‐FITC/PI cell apoptosis kit (4A biotech).^29^ After the treatment period, PC‐12 cells were harvested and washed with cold PBS. The cells were then resuspended in Annexin V binding buffer and stained with Annexin V‐FITC and PI according to the manufacturer's instructions. The stained cells were analyzed using a flow cytometer, and data were acquired for at least 10,000 events per sample.

### 2.8 Plasmid transfection

For plasmid transfection, PC‐12 cells were seeded in 6‐well culture plates at a density of 2 × 10^5^ cells per well and allowed to adhere overnight. The cells were then transfected with the desired plasmids expressing the target gene or control plasmids using a transfection reagent from Vazyme Biotech Co., Ltd. for DNA transfection. The transfection complex was prepared by diluting the plasmid DNA in serum‐free Opti‐MEM and mixing it with the transfection reagent. The mixture was added to the cells, and the transfection process was allowed to proceed for the recommended duration. Control cells were transfected with an empty vector or a non‐targeting plasmid.

### 2.9 Measurement of mitochondrial membrane potential (MMP)

Mitochondrial membrane potential (MMP) was assessed using JC‐1 kit (MCE),^30^ a fluorescent probe that exhibits a potential‐dependent dual emission. After the treatment period, PC‐12 cells were washed with pre‐warmed PBS and incubated with JC‐1 staining solution (5 μg/mL) in serum‐free DMEM at 37°C for 30 min in the dark. Subsequently, the cells were washed again with PBS to remove excess JC‐1 staining solution. To visualize the MMP changes, the stained cells were examined using a fluorescence microscope. In healthy cells with intact MMP, JC‐1 forms aggregates, emitting red fluorescence. Conversely, in cells with disrupted MMP, JC‐1 remains in the monomeric form, emitting green fluorescence. The fluorescence images were captured, and representative images were selected for further analysis using the ImageJ software (NIH). Additionally, MMP was detected by a flow cytometer. In brief, PC‐12 cells were harvested and resuspended in PBS. The fluorescence intensity of JC‐1 aggregates (red) and JC‐1 monomers (green) was measured. The ratio of green to red fluorescence was calculated as an indicator of MMP.

### 2.10 Hoechst/PI staining

Cell death was evaluated using Hoechst 33342/propidium iodide (PI) staining, a widely used method for distinguishing between live and dead cells.^31^ After the treatment period, PC‐12 cells were washed with PBS and incubated with Hoechst 33342 staining solution (10 μg/mL) and PI staining solution (5 μg/mL) for 15 minutes at room temperature in the dark. The stained cells were examined using a fluorescence microscope. Live cells displayed normal nuclear morphology with uniform Hoechst staining, while dead cells exhibited condensed and fragmented nuclei. Representative images were captured, and the percentage of cell death was determined based on the fluorescence intensity of cells stained with PI to Hoechst using the ImageJ software.

### 2.11 Western blotting

After the treatment period, PC‐12 cells were washed with ice‐cold PBS and lysed using an appropriate lysis buffer supplemented with protease and phosphatase inhibitors. The lysates were collected and centrifuged to obtain the protein‐containing supernatant. Protein concentrations in the supernatant were determined using the Bradford reagent. Equal amounts of protein samples were subjected to sodium dodecyl sulfate–polyacrylamide gel electrophoresis (SDS‐PAGE). The proteins were separated and electrophoresed under specific conditions. Following electrophoresis, the proteins were transferred from the gel onto a nitrocellulose or polyvinylidene difluoride (PVDF) membrane. The membrane was then blocked with 5% non‐fat milk to prevent nonspecific binding of antibodies. The blocked membrane was incubated overnight at 4°C with primary antibodies specific to the target proteins of interest. After washing, the membrane was incubated with secondary antibodies at room temperature for 1 hour. The immunoreactive protein bands on the membrane were visualized using UltraSignal Hypersensitive ECL Chemiluminescent Substrate (4A Biotech). The chemiluminescent signals were captured using a ChemiDoc MP Imaging System (Bio‐Rad). The band intensities were quantified using the ImageJ analysis software.

### 2.12 Maintenance of *C. elegans*

*C. elegans* strains, including N2 and transgenic strains, were obtained from the Caenorhabditis Genetics Center (CGC) or other reliable sources. The worms were maintained on nematode growth medium (NGM) agar plates seeded with *Escherichia coli* (*E. coli*) OP50 as a food source. To obtain a synchronized population of worms, adult hermaphrodites were allowed to lay eggs on NGM plates for 4 h. Subsequently, the adult worms were removed, and the plates containing eggs were incubated at 20°C until the worms reached the desired developmental stage.

### 2.13 Measurement of α‐synuclein in NL5901 worms

After the treatment period, NL5901 worms were anesthetized using sodium azide. The worms were mounted on agarose pads, and the fluorescence of GFP‐tagged α‐synuclein was visualized using a fluorescence microscope. Representative images were captured from multiple fields, ensuring a sufficient number of worms were analyzed. For quantitative analysis, the fluorescence intensity of GFP in individual worms was measured using the ImageJ software, and the MFI was calculated.

### 2.14 Assessment of motor function

Motor function was assessed by measuring the locomotion behavior of NL5901 worms.^32^ The synchronized L4 stage nematodes were cultured until the 5th or 10th day post‐synchronization. To perform the assay, a 35 mm petri dish was prepared by adding 1 mL of M9 buffer solution. An appropriate number of nematodes were carefully picked using a nematode picker and transferred to the petri dish containing the buffer solution. The nematodes were allowed to settle for 2 min to acclimate to the new environment. After the acclimation period, the nematodes were observed under a stereomicroscope. Within a 20 s interval, the number of body swings or thrashes made by each nematode was recorded.

### 2.15 Measurement of dopaminergic neurons

After the treatment period, BZ555 worms were immobilized using sodium azide. The worms were mounted on agarose pads, and the fluorescence of GFP‐labeled dopaminergic neurons was visualized using a fluorescence microscope. Representative images were captured from multiple fields, and the fluorescence intensity of GFP in individual worms was measured using the ImageJ software.

### 2.16 Food‐sensing ability

The food‐sensing ability of BZ555 worms was evaluated by observing their chemotaxis behavior toward food sources.^32^ Using a nematode picking needle, nematodes were randomly selected and transferred onto NGM plates. The nematodes were allowed to acclimate to the sterile environment for 2 min. Subsequently, the nematodes were observed under a microscope, and the number of movements exhibited within a 20‐second period was recorded. To determine the reduction rate of nematode food perception, the following formula was employed with reference to the previous method.^12^

### 2.17 Pharyngeal pumping assay

The pharyngeal pumping behavior of N2 worms was assessed by counting the number of contractions made by the pharyngeal muscles within a 20‐second period.^33^ The worms were transferred to agarose pads, and their pharyngeal pumping was observed using a stereomicroscope. The contractions were visually counted by an observer blinded to the treatment groups, and the pumping rate was calculated.

### 2.18 Measurement of reproductive fitness

The reproductive fitness of N2 worms was assessed by measuring brood size.^33^ The worms were individually transferred to new NGM plates and allowed to lay eggs per 24 h until the loss of reproductive ability. The number of offspring (brood size) produced by each worm was counted.

### 2.19 Lifespan assay

The lifespan of N2 worms was assessed by monitoring the survival of individual worms over time.^27^ The worms were transferred to fresh NGM plates at regular intervals (e.g., every 1 day) to prevent overcrowding and maintain optimal conditions. The number of live and dead worms was recorded daily until all the worms in a particular group had expired. For quantitative analysis, the survival curves were generated by plotting the percentage of surviving worms against time. The median lifespan, defined as the time at which 50% of the worms had expired, was determined for each treatment group and control group.

### 2.20 Lipofuscin determination

Lipofuscin, a fluorescent pigment associated with aging and cellular damage, was determined in N2 worms.^33^ The worms were immobilized using sodium azide and mounted on agarose pads. The fluorescence of lipofuscin was visualized using a fluorescence microscope. Representative images were captured from multiple fields, and the intensity of lipofuscin fluorescence was measured and quantified for individual worms.

### 2.21 Stress resistance assay

The stress resistance of N2 worms was assessed by subjecting the worms to various stressors known to induce physiological stress, such as heat stress, oxidative stress, or pathogenic bacteria.^33^ For heat stress, the worms were transferred to pre‐warmed NGM plates and incubated at 35°C. For oxidative stress, the worms were exposed to 30 mM H_2_O_2_. For pathogenic bacteria, the worms were exposed to *pseudomonas aeruginosa* (PA14). The survival and health of the worms under these stress conditions were recorded. The survival rate was calculated as the percentage of worms that survived the stress exposure compared to the initial population.

### 2.22 Determination of ROS in worms

The ROS levels in worms were determined using the DHE reagent. In brief, after treatment, the worms were incubated with the DHE solution, and representative images were captured by a fluorescence microscope. The fluorescence intensity was measured and quantified for individual worms using the ImageJ software.

### 2.23 RNAi feeding assay

To investigate the functional role of specific genes in *C. elegans*, worms were subjected to RNAi by feeding them with bacteria expressing double‐stranded RNA (dsRNA) targeting the respective genes.^21^ The synchronized NL5901 and BZ555 worms were transferred to NGM plates seeded with *mpk‐1* RNAi bacteria and allowed to grow until the desired stage or age for subsequent experiments. Control worms were cultured on NGM plates seeded with HT115 bacteria.

### 2.24 Statistical analysis

Data were expressed as the mean ± standard deviation (SD) and were derived from at least three independent experiments. Analyses were conducted using GraphPad Prism 8.0 software. The normality of the data was ascertained using the Shapiro–Wilk test, while the Brown–Forsythe test was used to check for the homogeneity of variances. The Kaplan–Meier method was employed to evaluate the lifespan, paralysis, survival rate, and stress resistance parameters. The significance of differences was determined using the log‐rank test to compute *p*‐values. For comparisons among multiple groups, a one‐way analysis of variance (ANOVA) followed by post hoc Tukey's test was used, and a two‐way ANOVA was applied when appropriate. A *p*‐value of less than 0.05 was considered statistically significant.

## 3 RESULTS

### 3.1 CFE protects PC‐12 cells against H_2_O_2_ ‐induced cytotoxicity

**FIGURE 1:**
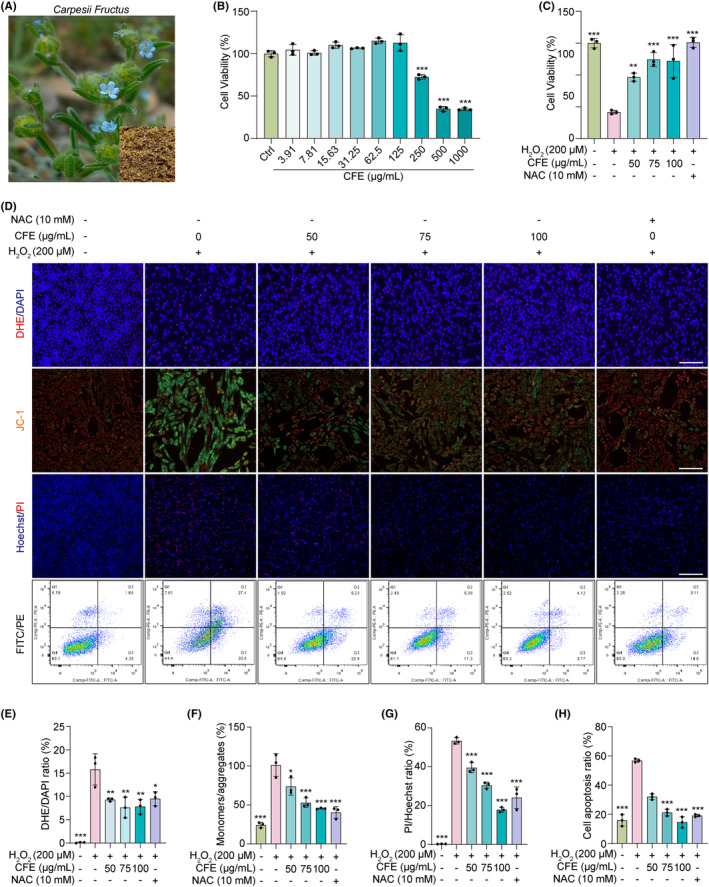
CFE protects PC‐12 cells against H_2_O_2_‐induced cytotoxicity. (A) The dry Common Carpesium Fruit and the plant from which it originates. (B) The bar chart indicates the cell viability of PC‐12 cells treated with CFE at the indicated concentrations. (C) The bar chart indicates the cell viability of PC‐12 cells treated with H_2_O_2_ in the presence or absence of CFE and NAC at the indicated concentrations. (D) PC‐12 cells were treated with H_2_O_2_ in the presence or absence of CFE and NAC at the indicated concentrations. After treatment, representative images of DHE staining, JC‐1 staining, and Hoechst/PI staining were captured by fluorescence microscopy. Magnification: 20x for DHE staining and JC‐1 staining and 10x for Hoechst/PI staining, scale bar: 100 μm for DHE staining and JC‐1 staining and 200μm for Hoechst/PI staining. Meanwhile, cell apoptosis was detected by flow cytometry using the FITC/PE apoptosis detection kit. (E–H) Bar charts indicate the DHE/DAPI ratio, JC‐1 monomers/aggregates, PI/Hoechst ratio, and cell apoptosis ratio of PC‐12 cells. Error bars, S.D., *p < 0.05; **p < 0.01; ***p < 0.001.

Oxidative stress has emerged as a critical pathological feature of PD, implicating the imbalance between reactive oxygen species (ROS) and antioxidant defense mechanisms in the neurodegenerative process, shedding light on potential therapeutic strategies to mitigate disease progression.^34^ In this study, we employed H_2_O_2_‐induced PC‐12 cells and investigated the neuroprotective effect of CFE, which is the ethanol extract of *Carpesii fructus* (Figure 1A). First, the constituents in CFE were identified using UHPLC‐DAD‐Q/TOF‐MS/MS. Figure S1 and Table S1 show that 17 main constituents were characterized based on the literature^35^ and the Dictionary of Natural Products (DNP) database, accessible at https://dnp.chemnetbase.com. After examining the cytotoxicity of CFE and H_2_O_2_ in PC‐12 cells (Figure 1B and S2), we investigated the effect of CFE at concentrations of 50–100 μg/mL on the inhibition of H_2_O_2_‐induced cell death. The results in Figure S3 and Figure 1C–E demonstrate that CFE dose‐dependently improved cell morphology, increased cell viability, and reduced cell death in H_2_O_2_‐induced PC‐12 cells. Furthermore, we evaluated mitochondrial damage by measuring the mitochondrial membrane potential (MMP) using JC‐1 staining and ROS production using DHE staining. Figures 1D,F, and S2 show that CFE significantly restored MMP, as evidenced by the decreased ratio of JC‐1 monomers (green fluorescence) to JC‐1 aggregates (red fluorescence). Moreover, Figures 1D,G, and S4 illustrate that CFE reduced ROS production, as evidenced by the DHE intensity (red fluorescence). Additionally, we examined the inhibitory effect of CFE on H_2_O_2_‐induced cell apoptosis, and the results in Figure 1D,H showed that CFE remarkably decreased the apoptosis rate of H_2_O_2_‐induced PC‐12 cells. Collectively, the above data suggest that CFE protects PC‐12 cells against H_2_O_2_‐induced cytotoxicity, possibly by inhibiting mitochondria‐mediated apoptosis.

### 3.2 CFE protects PC‐12 cells against 6‐OHDA‐induced cytotoxicity

**FIGURE 2:**
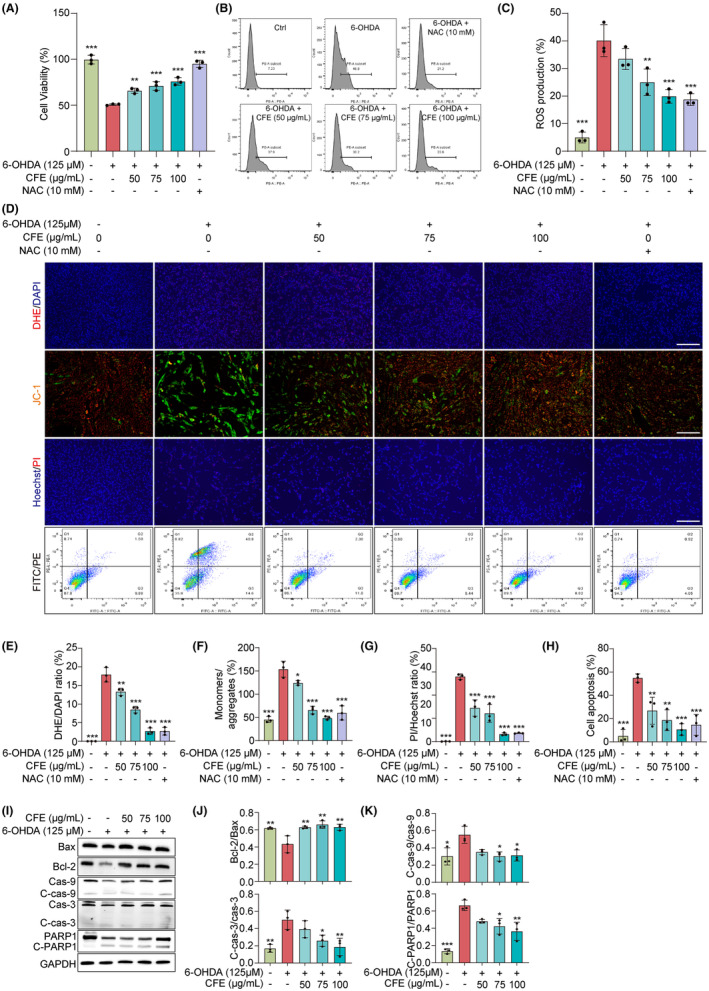
CFE protects PC‐12 cells against 6‐OHDA‐induced cytotoxicity. (A) The bar chart indicates the cell viability of PC‐12 cells treated with 6‐OHDA in the presence or absence of CFE and NAC at the indicated concentrations. (B) Representative flow cytometry images showing the DHE intensity of PC‐12 cells treated with 6‐OHDA in the presence or absence of CFE and NAC at the indicated concentrations. (C) The bar chart indicates the ROS production in PC‐12 cells. (D) PC‐12 cells were treated with 6‐OHDA in the presence or absence of CFE and NAC at the indicated concentrations. After treatment, representative images of DHE staining, JC‐1 staining, and Hoechst/PI staining were captured by fluorescence microscopy. Magnification: 20x for JC‐1 staining and DHE staining and 10x for Hoeschst/PI staining, scale bar: 100 μm for JC‐1 staining and DHE staining and 200 μm for Hoechst/PI staining. Meanwhile, cell apoptosis was detected by flow cytometry using the FITC/PE apoptosis detection kit. (E‐H) Bar charts indicate the DHE/DAPI ratio, JC‐1 monomers/aggregates, PI/Hoechst ratio, and cell apoptosis ratio of PC‐12 cells. (I) Representative Western blotting images of Bax, Bcl‐2, Cas‐9, C‐cas‐9, Cas‐3, C‐cas‐3, PARP1, C‐PARP1, and GAPDH in 6‐OHDA‐induced PC‐12 cells treated with or without CFE at the indicated concentrations. Full unedited gel/blots are provided in Figure S11, where the protein molecular weight markers were labeled. (J‐K) Bar charts indicate the ratio of Bcl‐2/Bax, C‐cas‐9/Cas‐9, C‐cas‐3/Cas‐3, and C‐PARP1/PARP1. Error bars, S.D., *p < 0.05; **p < 0.01; ***p < 0.001.

The 6‐OHDA‐induced model of PD has provided valuable insights into the pathogenesis of dopaminergic neuronal degeneration, offering a versatile tool to investigate molecular mechanisms and explore neuroprotective interventions.^22^ In this study, we investigated the neuroprotective effect of CFE in 6‐OHDA‐induced PC‐12 cells after examining the cytotoxicity of 6‐OHDA in PC‐12 cells (Figure S5). The results in Figures 2A,D,E, and S6 show that CFE significantly increased cell viability, reduced cell death, and improved cell morphology in 6‐OHDA‐induced PC‐12 cells. Then, we examined mitochondrial damage by measuring the mitochondrial membrane potential (MMP) using JC‐1 staining and reactive oxygen species (ROS) production using DHE staining. Figures 2D,F, and S7 show that CFE significantly decreased the ratio of JC‐1 monomers to JC‐1 aggregates, suggesting a restoration of MMP in 6‐OHDA‐induced PC‐12 cells. Additionally, Figure 2B–D,G shows that CFE remarkably attenuated the DHE intensity, indicating a reduction in ROS production. Furthermore, CFE inhibited 6‐OHDA‐induced apoptosis in PC‐12 cells, as evidenced by the decreased apoptosis rate (Figure 2D,H) and the expression of apoptosis‐related proteins (Figure 2I–K). Thus, CFE protects PC‐12 cells against 6‐OHDA‐induced cytotoxicity.

### 3.3 CFE reduces α‐synuclein expression in PC‐12 cells

**FIGURE 3:**
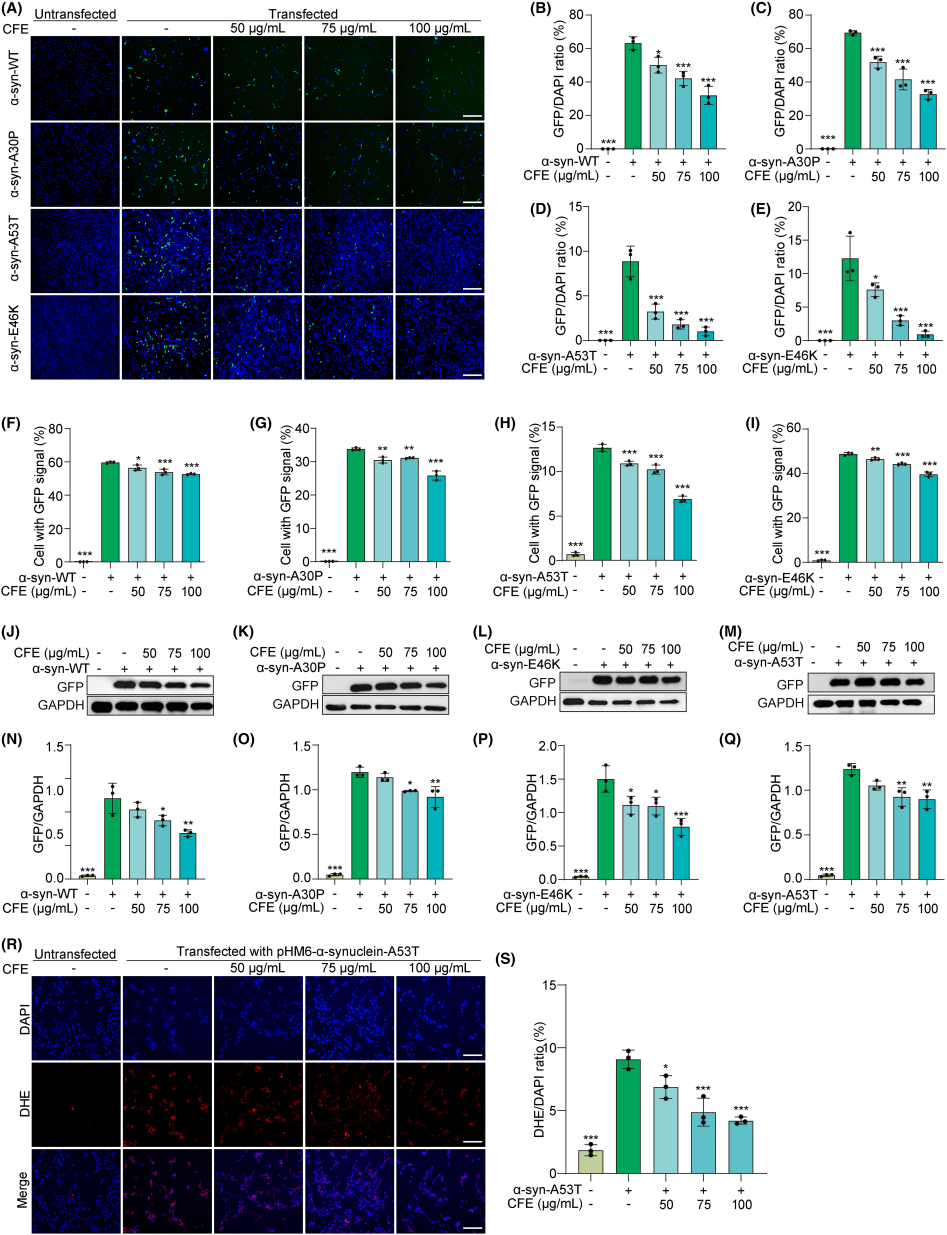
CFE reduces α‐synuclein expression in PC‐12 cells. (A) Representative images of EGFP‐α‐synuclein‐WT‐, EGFP‐α‐synuclein‐A53T‐, EGFP‐α‐synuclein‐A30P‐, or EGFP‐α‐synuclein‐E46K‐transfected PC‐12 cells treated with CFE at the indicated concentrations. Magnification: 10x, scale bar: 200 μm. (B‐E) Bar charts indicate the GFP/DAPI ratio in PC‐12 cells. (F‐I) Bar charts indicate the percentage of cells with GFP signal, which was determined by flow cytometer in Figure S9. (J‐M) Representative Western blotting images of GFP and GAPDH in EGFP‐α‐synuclein‐WT‐, EGFP‐α‐synuclein‐A53T‐, EGFP‐α‐synuclein‐A30P‐, or EGFP‐α‐synuclein‐E46K‐transfected PC‐12 cells treated with CFE at the indicated concentrations. Full unedited gel/blots are provided in Figure S12, where the protein molecular weight markers were labeled. (N‐Q) Bar charts indicate the ratio of GFP/GAPDH. (R) Representative images of pHM6‐α‐synuclein‐A53T‐transfected PC‐12 cells stained with DHE reagent and treated with CFE at the indicated concentrations. Magnification: 20x, scale bar: 100 μm. (S) The bar chart indicates the DHE/DAPI ratio in PC‐12 cells. Error bars, S.D., *p < 0.05; **p < 0.01; ***p < 0.001.

α‐synuclein, a protein abundantly expressed in neurons, has emerged as a key player in the pathogenesis of PD, with its abnormal aggregation and deposition contributing to the formation of Lewy bodies and the subsequent neurodegenerative cascade.^12^ In this study, we employed and transiently transfected plasmids, including EGFP‐N1, EGFP‐α‐synuclein‐WT, EGFP‐α‐synuclein‐A53T, EGFP‐α‐synuclein‐A30P, and EGFP‐α‐synuclein‐E46K into PC‐12 cells. Then, we determined the expression of α‐synuclein using fluorescence microscopy, flow cytometry, and Western blotting methods. Figure S8 and Figure 3A–E demonstrate that CFE had no effect on the transfection efficiency but significantly decreased the intensity of GFP. This decrease reflects the expression of EGFP‐α‐synuclein‐WT, EGFP‐α‐synuclein‐A53T, EGFP‐α‐synuclein‐A30P, and EGFP‐α‐synuclein‐E46K, respectively. Flow cytometry analysis results revealed that CFE reduced the percentage of cells with GFP signal in transfected PC‐12 cells, suggesting CFE reduced α‐synuclein expression (Figure S8 and Figure 3F–I). Additionally, we employed a GFP antibody to determine the protein expression of GFP, and Figure 3J–Q demonstrated a significant reduction in GFP expression in these transfected PC‐12 cells treated with CFE at indicated concentrations. Furthermore, we determined the inhibitory effect of CFE on ROS production in CFE‐treated pHM6‐α‐synuclein‐A53T‐overexpressing PC‐12 cells, and Figure 3R,S displays that CFE remarkably reduced the intensity of DHE in PC‐12 cells, suggesting a reduction in ROS production. Collectively, CFE reduces α‐synuclein expression and its induced ROS production.

### 3.4 CFE inhibits 6‐OHDA‐induced cell death by regulating the MAPK signaling pathway in PC‐12 cells

**FIGURE 4:**
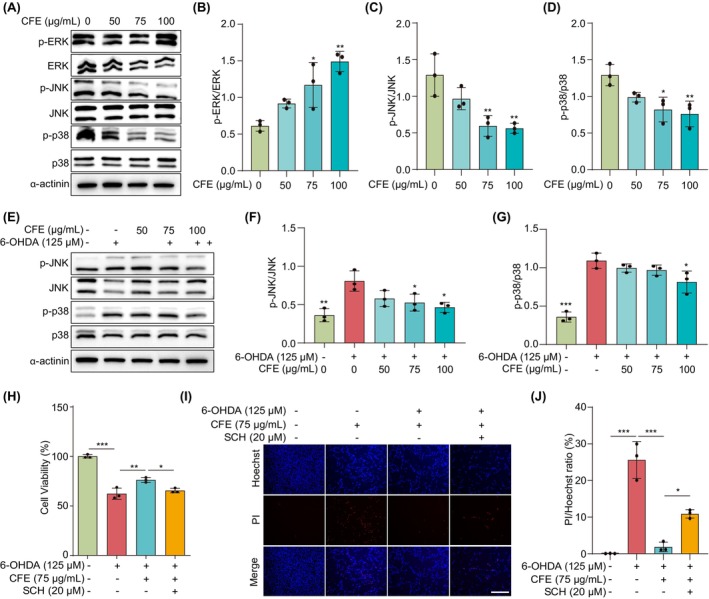
CFE inhibits 6‐OHDA‐induced cell death by regulating the MAPK signaling pathway in PC‐12 cells. (A) Representative Western blotting images of p‐ERK, ERK, p‐JNK, JNK, p‐p38, p38, and β‐Actin in PC‐12 cells treated with CFE at the indicated concentrations. Full unedited gel/blots are provided in Figure S13, where the protein molecular weight markers were labeled. (B–D) Bar charts indicate the ratio of p‐ERK/ERK, p‐JNK/JNK, and p‐p38/p38. (E) Representative Western blotting images of p‐JNK, JNK, p‐p38, p38, and β‐Actin in 6‐OHDA‐treated PC‐12 cells with or without CFE at the indicated concentrations. Full unedited gel/blots are provided in Figure S13, where the protein molecular weight markers were labeled. (F,G) Bar charts indicate the ratio of p‐JNK/JNK and p‐p38/p38. (H) The bar chart indicates the cell viability of 6‐OHDA‐treated PC‐12 cells with or without CFE and SCH at the indicated concentrations. (I) Representative Hoechst/PI staining images of 6‐OHDA‐treated PC‐12 cells with or without CFE and SCH at the indicated concentrations. Magnification: 10x, Scale bar: 200 μm. (J) The bar chart indicates the ratio of Hoechst/PI. Error bars, S.D., *p < 0.05; **p < 0.01; ***p < 0.001.

In the context of neurodegeneration in PD, aberrant MAPK signaling has been implicated as a key mediator of neuronal death in response to diverse insults such as 6‐OHDA.^36^ In this study, we employed Western blotting to investigate the mechanism of action of CFE. Figure 4A–D shows that CFE dose‐dependently upregulated ERK but downregulated JNK and p38 signaling pathways. In 6‐OHDA‐induced PC‐12 cells, the ratio of p‐JNK/JNK and p‐p38/p38 was significantly increased, where CFE treatment successfully reversed these increasing trends (Figure 4E–G). The above data suggest that CFE regulates the MAPK signaling pathways. Furthermore, we employed SCH772984, a specific inhibitor of ERK, to cotreat with CFE, and we found that CFE counteracted the effect of SCH772984 on the improvement of cell viability and the inhibition of cell death (Figure 4H–J). Collectively, CFE exhibits a neuroprotective effect in 6‐OHDA‐induced PC‐12 cells via regulating the MAPK signaling pathway.

### 3.5 CFE mitigates α‐synuclein pathology in the NL5901 strain

**FIGURE 5:**
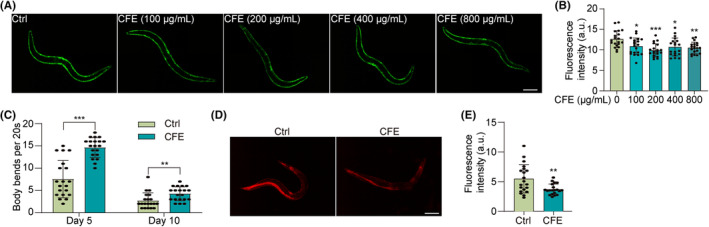
CFE mitigates α‐synuclein pathology in the NL5901 strain. (A) Representative images of GFP‐α‐synuclein in the muscle wall of NL5901 worms treated with CFE at the indicated concentrations. Magnification: 10x, scale bar: 200 μm. (B) The bar chart indicates the GFP intensity in NL5901 worms. (C) The bar chart indicates the number of body bends of NL5901 worms treated with CFE on Day 5 and Day 10. (D) Representative DHE staining images of CFE‐treated NL5901 worms. Magnification: 10x, scale bar: 200 μm. (E) The bar chart indicates the DHE intensity in NL5901 worms. Error bars, S.D., *p < 0.05, **p < 0.01, ***p < 0.001, n = 20.

To investigate the neuroprotective effect of CFE in vivo, we employed NL5901 strain‐expressing α‐synuclein in the body wall muscle. Figure 5A,B shows that CFE significantly reduced the expression of α‐synuclein, as evidenced by reduced GFP intensity. Additionally, we examined the motility of NL5901 worms, and Figure 5C shows that CFE significantly increased the body bends at days 5 and 10, suggesting an improvement in motility. Furthermore, we measured the ROS production in NL5901 worms, and Figure 5D,E shows that CFE decreased the DHE intensity in worms, indicating a reduction in ROS production. Collectively, CFE alleviates α‐synuclein pathology in the NL5901 strain.

### 3.6 CFE inhibits the degeneration of dopaminergic neurons in BZ555 strain

**FIGURE 6:**
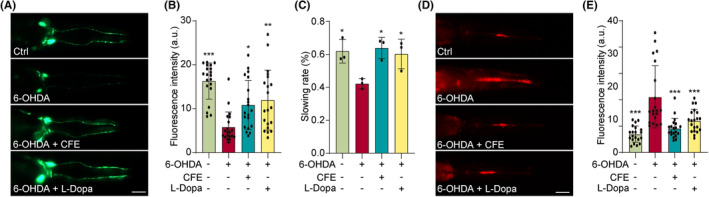
CFE prevents the degeneration of dopaminergic neurons in the BZ555 strain. (A) Representative images showing the GFP expression in DA neurons of 6‐OHDA‐treated BZ555 worms with or without CFE and L‐Dopa. Magnification: 20x, scale bar: 100 μm. (B) The bar chart indicates the GFP intensity in BZ555 worms. *n* = 20. (C) The bar chart indicates the slowing rate of 6‐OHDA‐treated BZ555 worms with or without CFE and L‐Dopa. *n* = 20. (D) Representative DHE staining images of 6‐OHDA‐treated BZ555 worms with or without CFE and L‐Dopa. Magnification: 20x, scale bar: 100 μm. (E) The bar chart indicates the DHE intensity in BZ555 worms. *n* = 20. Error bars, S.D., *p < 0.05, **p < 0.01, ***p < 0.001, *n* = 20.

The BZ555 strain, a genetically modified model organism, has emerged as a valuable tool in the study of dopaminergic neurons in PD research. In this study, we examined the viability of dopaminergic neurons in 6‐OHDA‐induced BZ555 worms with or without CFE. Figure 6A,B demonstrates a progressive loss of dopaminergic neurons, reflected by the reduced GFP signal in the anterior cephalic neurons (CEPs). However, the treatment with CFE or L‐Dopa successfully restored the expression of GFP, suggesting that CFE and L‐Dopa rescue the dopaminergic neurons in 6‐OHDA‐induced BZ555 worms. Then, we investigated the improvement effect of CFE on the food‐sensing ability, and Figure 6C shows that CFE and L‐Dopa significantly increased the slowing rate of 6‐OHDA‐induced BZ555 worms. Additionally, we detected the ROS production in BZ555 worms using DHE staining. Figure 6D,E demonstrates an obvious increase in fluorescence intensity in 6‐OHDA‐induced BZ555 worms, whereas the CFE or L‐Dopa treatment significantly inhibited the DHE intensity. Collectively, CFE inhibits the degeneration of dopaminergic neurons in BZ555 worms.

### 3.7 CFE extends lifespan and enhances healthy aging in *C*. *elegans*

**FIGURE 7:**
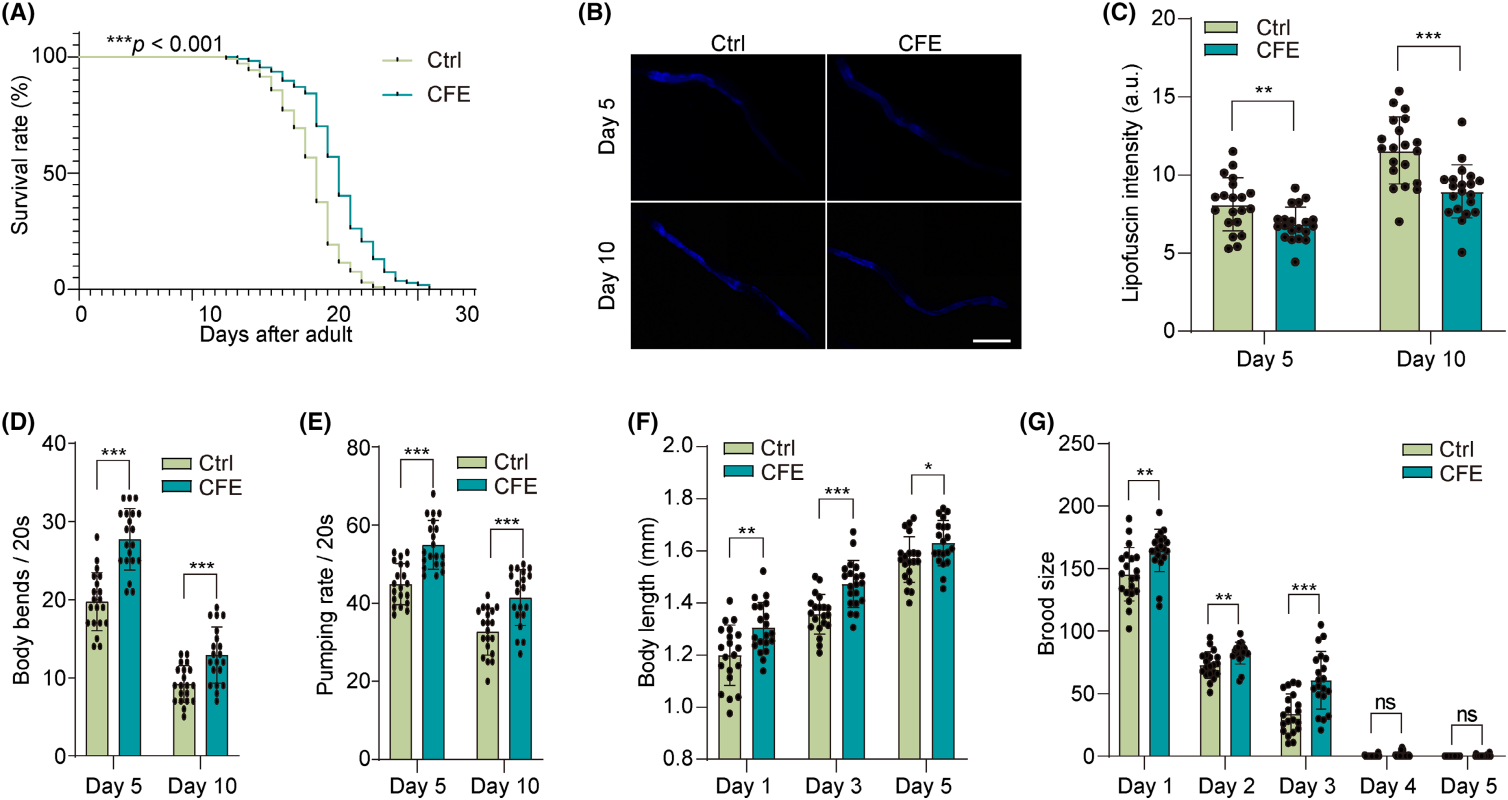
CFE extends lifespan and enhances healthy aging in *C. elegans*. (A) The survival curve of N2 worms treated with or without CFE. (B) Representative images showing the accumulation of lipofuscin in N2 worms treated with CFE at Day 5 and Day 10. Magnification: 20x, scale bar: 100 μm. (C) The bar chart indicates the blue fluorescence intensity reflecting the lipofuscin accumulation in N2 worms. (D) The bar chart indicates the number of body bends within 20 s of N2 worms treated with or without CFE on Day 5 and Day 10. (E) The bar chart indicates the pumping rate within 20 s of N2 worms treated with or without CFE at Day 5 and Day 10. (F) The bar chart indicates the body length of N2 worms treated with or without CFE at Day 1, Day 3, and Day 5. (G) The bar chart indicates the brood size of N2 worms treated with or without CFE at Day 1, Day 2, Day 3, Day 4, and Day 5. Error bars, S.D., *p < 0.05, **p < 0.01, ***p < 0.001, *n* = 20.

Aging represents a key determinant in the pathogenesis of PD.^37^ The multifaceted role of aging involves complex interplays of proteostasis decline, mitochondrial dysfunction, neuroinflammation, and cellular senescence, culminating in the selective vulnerability of dopaminergic neurons. In this study, we investigated the effect of CFE on extending aging and improvement of aging‐related phenotypes in N2 worms. Figure 7A shows that CFE significantly prolonged the lifespan of N2 worms, as evidenced by the increased survival rate. The accumulation of lipofuscin is recognized as a marker of aging.^33^ With aging, the intensity of blue fluorescence reflecting the content of lipofuscin increases. However, CFE treatment distinctly reduced the intensity of blue fluorescence (Figure 7B,C). In addition, the motility decreases with aging, and CFE treatment improved motility, as evidenced by the increased body bends within 20 s (Figure 7D). Studies have shown that decreased pharyngeal pumping rate and diet restriction are correlated with the extension of aging in nematodes.^38^ Here, we found that the pharyngeal pumping rate of worms increased at Day 5 and Day 10 after CFE treatment, suggesting that the anti‐aging effect of CFE is independent of diet restriction (Figure 7E). Furthermore, the reproductive ability of worms upon CFE treatment was evaluated. As shown in Figure 7F, G, CFE significantly increased the body length and brood size of N2 worms, suggesting that CFE may have non‐reproductive toxicity and promote the growth and development of worms. Collectively, CFE prolongs lifespan and promotes healthy aging in *C. elegans*.

### 3.8 CFE enhances the stress resistance of *C. elegans*

**FIGURE 8:**
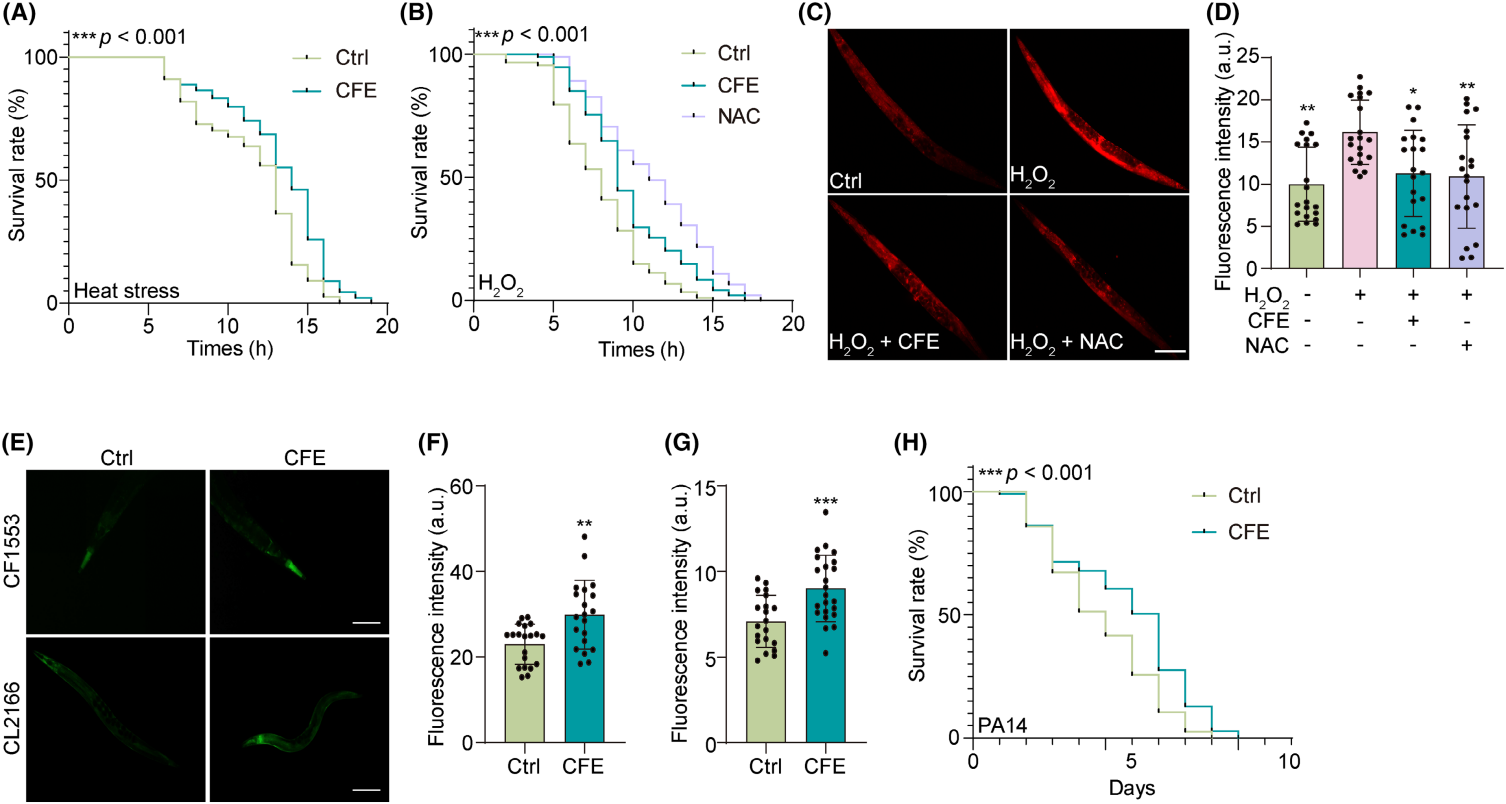
CFE enhances the stress resistance of *C. elegans*. (A) The survival curve of CFE‐treated N2 worms when the temperature was shifted from 25°C to 35°C. (B) The survival curve of H_2_O_2_‐treated N2 worms with or without CFE and NAC. (C) Representative DHE staining images of H_2_O_2_‐treated N2 worms with or without CFE and NAC. Magnification: 10x, scale bar: 200 μm. (E) The bar chart indicates the DHE intensity in N2 worms. (C) Representative images showing the expression of GFP‐sod‐3 in CFE‐treated CF1553 worms (Magnification: 20x, scale bar: 100 μm) and the expression of GFP‐gst‐4 in CFE‐treated CL2166 worms (Magnification: 10x, scale bar: 200 μm). (F) The bar chart indicates the expression of GFP‐sod‐3 in CF1553 worms. (G) The bar chart indicates the expression of GFP‐gst‐4 in CF1553 worms. (H) The survival curve of PA14‐treated N2 worms with or without CFE. Error bars, S.D., *p < 0.05; **p < 0.01; ***p < 0.001, *n* = 20.

*C. elegans* serves as an exceptional model to study stress resistance during aging.^33^ In this study, we examined the effect of CFE on the resistance of worms to various stresses, including heat stress, oxidative stress, and pathogenic bacteria. Figure 8A shows that CFE significantly increased the survival rate of N2 worms when the incubation temperature was shifted from 25°C to 35°C. Under the exposure of 30 mM H_2_O_2_, the treatment of CFE and NAC remarkably increased the survival rate of N2 worms (Figure 8B). Concurrently, the DHE staining images displayed that CFE and NAC reduced the intensity of fluorescence in N2 worms (Figure 8C,D). Additionally, we examined the expression of sod‐3 and gst‐4, two important antioxidative genes. Figure 8E–G shows that CFE significantly increased the intensity of GFP, reflecting the expression of sod‐3 in CF1553 strain and gst‐4 in CL2166 strain, respectively. Therefore, CFE exhibits an antioxidative effect in worms. Furthermore, CFE treatment could increase the survival rate of N2 worms treated with PA14, suggesting that CFE resists pathogenic bacteria (Figure 8H). Taken together, CFE increases the stress resistance of *C. elegans*.

### 3.9 CFE exhibits anti‐PD effects in *C. elegans* by activating *mpk‐1*

**FIGURE 9:**
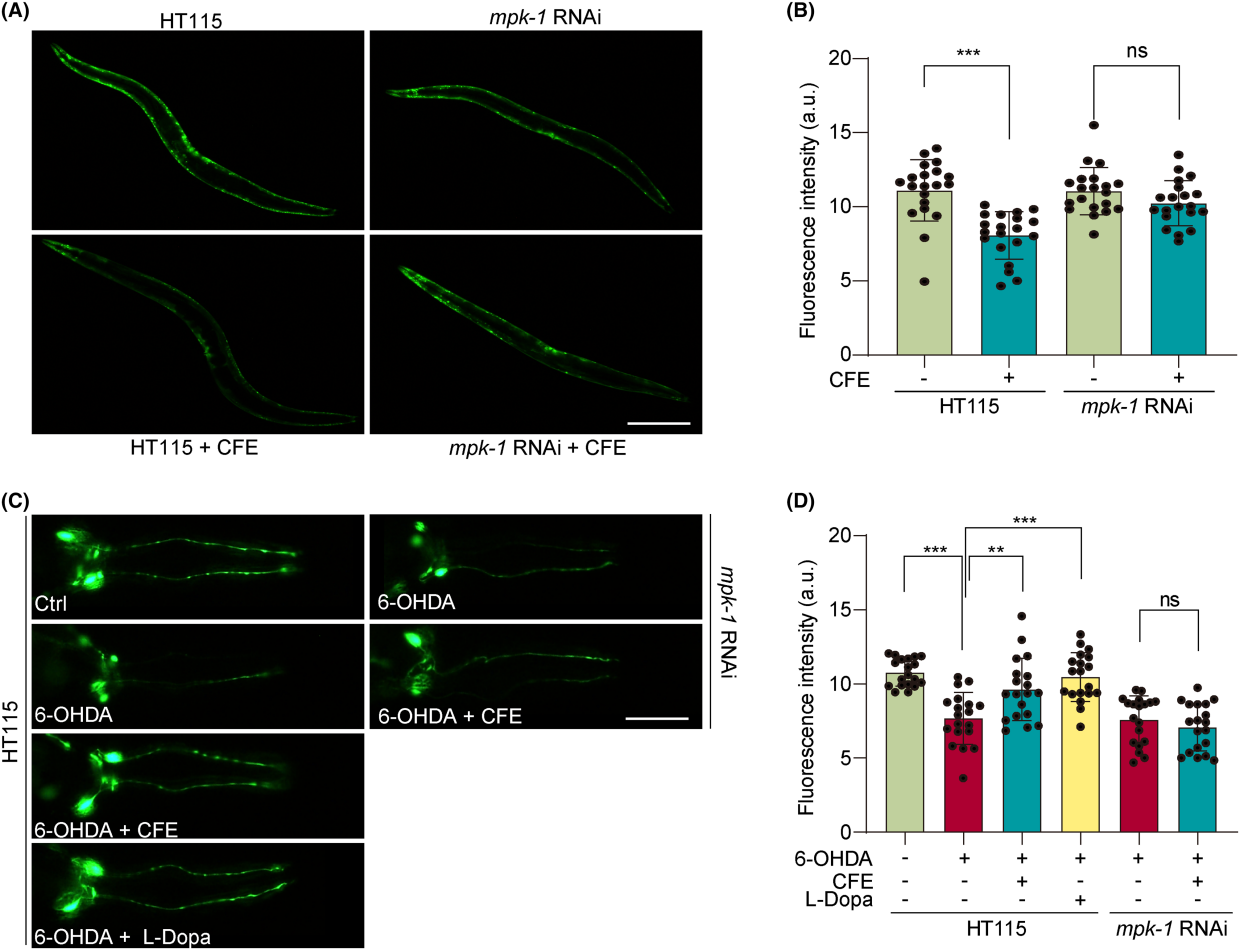
CFE exhibits anti‐PD effects in *C. elegans* by activating *mpk‐1*. (A) Representative images show the expression of GFP‐α‐synuclein in CFE‐treated NL5901 worms fed with HT115 or *mpk‐1* RNAi bacteria. Magnification: 10×, scale bar: 200 μm (B) The bar chart indicates the GFP intensity in NL5901 worms. (C) Representative images display the GFP expression in DA neurons of 6‐OHDA‐treated BZ555 worms with or without CFE and L‐Dopa, fed with HT115 or *mpk‐1* RNAi bacteria. Magnification: 20×, scale bar: 100 μm. (D) The bar chart indicates the GFP intensity in BZ555 worms. Error bars, S.D., **p < 0.01; ***p < 0.001, *n* = 20.

As a member of the MAPK family, *mpk‐1* plays a crucial role in transducing extracellular signals to regulate diverse cellular processes, including development, stress response, and longevity.^39^ Its functional homology to mammalian ERK makes *mpk‐1* an essential and well‐studied component in understanding signaling pathways and cellular behaviors in *C. elegans*. In this study, we fed worms with *mpk‐1* RNAi bacteria to knock down the expression of *mpk‐1* in NL5901 and BZ555 worms. As shown in Figure 9A,B, the feeding of *mpk‐1* RNAi bacteria significantly reversed the inhibitory effect on the expression of α‐synuclein, as evidenced by the increased intensity of GFP. Additionally, the treatment of CFE and L‐Dopa remarkably restored the viability of dopaminergic neurons in 6‐OHDA‐induced BZ555 worms, whereas the feeding of *mpk‐1* RNAi bacteria attenuated the beneficial effect of CFE on dopaminergic neurons (Figure 9C,D). Collectively, CFE inhibits α‐synuclein expression and the degeneration of dopaminergic neurons via activating m*pk‐1*.

## 4 DISCUSSION

PD is a neurological disorder that primarily affects movement, and while it is a challenging condition, there have been significant advancements in understanding its pathological mechanisms and treatment options. To date, researchers have made substantial progress in unraveling the pathological mechanism of PD. It is primarily characterized by the loss of dopamine‐producing neurons in the brain's substantia nigra region. This dopamine deficiency leads to the motor symptoms we commonly associate with Parkinson's, such as tremors, bradykinesia, and rigidity. At present, various therapeutic approaches include medications that help replenish dopamine levels,^40^ deep brain stimulation (DBS),^41^ and even promising gene therapies.^42^ These treatments have brought significant relief to many patients and have improved their quality of life. While there have been some advancements, PD still lacks a definitive cure.^43^ Some treatments offer symptomatic relief, but they do not address the root cause of the disease. In addition, not all patients respond well to current treatments, and the side effects of certain medications can be quite problematic. Also, long‐term use of medications can lead to complications, making it difficult for patients to manage the disease effectively.^44^ Moreover, the pathological mechanism of PD is not entirely understood, and there may be other factors contributing to its development that we have not discovered yet. Until we have a comprehensive understanding, finding a lasting cure will remain a challenge.

Natural medicines such as traditional herbal medicine have been used for centuries in various cultures to address a wide range of health conditions. Traditional herbal medicine often relies on plant‐based compounds that may possess therapeutic properties. Some herbs have shown potential anti‐inflammatory, antioxidant, and neuroprotective effects, which could be beneficial for managing the symptoms of Parkinson's disease.^13^ Oxidative stress refers to an imbalance between the production of ROS and the body's ability to neutralize them with antioxidants. When this balance is disrupted, an excessive amount of ROS accumulates, leading to cellular damage and dysfunction.^45^ Numerous studies have shown that oxidative stress represents a common thread that connects various pathological features of PD, such as mitochondrial dysfunction,^46^ α‐synuclein aggregation,^47^ environmental toxins,^48^ and glia activation.^49^ In this study, we employed H_2_O_2_‐induced PC‐12 cells to screen the natural product library and found that CFE could significantly protect PC‐12 cells against H_2_O_2_‐induced cytotoxicity. This effect is likely mediated by CFE's ability to restore mitochondrial membrane potential (MMP) and reduce ROS production, thus inhibiting mitochondria‐mediated apoptosis. These findings suggest a potential therapeutic strategy for mitigating oxidative stress‐induced neurodegeneration in PD using CFE. Then, we investigated the neuroprotective effect of CFE against 6‐OHDA‐induced cytotoxicity in PC‐12 cells. The data reveal that CFE effectively increased cell viability, reduced cell death, and attenuated mitochondrial damage and ROS production. Furthermore, CFE's ability to inhibit 6‐OHDA‐induced apoptosis in PC‐12 cells highlights its potential as a promising neuroprotective agent against dopaminergic neuronal degeneration in PD. Emerging evidence suggests that α‐synuclein aggregation and deposition are key pathological features in PD. We examined the impact of CFE on reducing α‐synuclein expression in PC‐12 cells transfected with various α‐synuclein mutants. Our results indicate that CFE significantly reduces α‐synuclein expression and its induced ROS production, suggesting a potential therapeutic role in ameliorating α‐synuclein pathology in PD. Intriguingly, despite the neuroprotective effects observed with CFE treatment, our supplementary investigations revealed that CFE does not activate the autophagy–lysosome pathway (ALP) or the ubiquitin–proteasome system (UPS), both of which are pivotal in α‐synuclein degradation (Figure S10). This suggests that the protective mechanisms afforded by CFE in PD models might operate independently of these conventional protein degradation pathways. While many neuroprotective agents exert their effects by modulating ALP and UPS,^50^, ^51^ our findings emphasize the potential for diverse, multifaceted mechanisms of action of therapeutic compounds. The exact molecular underpinnings through which CFE mediates its effects warrant further exploration, particularly to unveil potential pathways or targets that might be uniquely modulated by this extract. It is noteworthy to mention the intricate relationship between α‐synuclein and oxidative stress. Our findings suggest that CFE may potentially disrupt this harmful cycle. By targeting the oxidative stress, the extract may indirectly mitigate α‐synuclein aggregation and its associated neurotoxicity. Understanding the interplay between α‐synuclein and oxidative stress can pave the way for developing more targeted therapeutic interventions for PD in the future. The MAPK pathway, encompassing ERK, JNK, and p38 MAPK cascades, is integral in transducing extracellular signals into cellular responses.^52^ Various extrinsic and intrinsic stressors can activate these cascades, leading to a series of phosphorylation events that regulate cellular activities.^52^ Emerging evidence suggests that the regulation of the MAPK signaling pathway is critical in PD.^53^ The dysregulation of MAPK pathways, particularly JNK and p38, has been associated with neurodegeneration and disease progression in response to neurotoxic insults.^54^ Therapeutic approaches that target specific components of the MAPK signaling cascade may hold promise for mitigating the pathological processes and providing neuroprotective effects in PD.^54^ Thus, to gain insight into the molecular mechanisms underlying CFE's neuroprotective effects, we explored the involvement of the MAPK signaling pathway. The data demonstrated that CFE could regulate the MAPK signaling pathways, particularly upregulating ERK and downregulating JNK and p38 signaling. The bioactive components within CFE might interface with upstream regulators or direct members of the MAPK cascade, providing neuroprotection by fostering cellular resilience against oxidative stress and apoptosis. Therefore, this modulation of MAPK signaling is likely contributing to CFE's neuroprotective effects in 6‐OHDA‐induced PC‐12 cells. To validate the neuroprotective effects of CFE in vivo, we employed *C. elegans* models, including NL5901 and BZ555 strains. The results suggest that CFE effectively alleviates α‐synuclein pathology in the NL5901 strain and inhibits the degeneration of dopaminergic neurons in the BZ555 strain, demonstrating its potential therapeutic utility against α‐synuclein‐associated neurodegeneration in PD. Moreover, we investigated the effects of CFE on aging‐related phenotypes in N2 worms. The results showed that CFE prolonged lifespan, reduced lipofuscin accumulation, improved motility, and promoted growth and development, indicating a positive impact on healthy aging in *C. elegans*. Furthermore, CFE enhanced the survival rates of *C. elegans* under various stress conditions, including heat stress, oxidative stress, and pathogenic bacteria exposure, indicating its potential as a protective agent against stress‐induced neurodegeneration in PD. At the same time, the feeding of RNAi bacteria demonstrated that the neuroprotective effects of CFE against α‐synuclein expression and dopaminergic neuronal degeneration are mediated through the activation of *mpk‐1*.

In interpreting the findings of our study, several key aspects warrant consideration. While our cellular and *C. elegans* models have yielded insightful data about the neuroprotective effects of CFE, it is vital to acknowledge the inherent limitations of these simplified systems; they capture specific facets of PD but may not fully mirror the multifaceted pathophysiology observed in humans. Consequently, the promising effects of CFE observed here underscore the need for subsequent investigations in mammalian models, which, if consistent, could pave the way for potential clinical applications in PD therapy. Additionally, the complex nature of CFE, comprising a myriad of bioactive molecules, necessitates a deeper dive into isolating and characterizing its active ingredients. Unraveling the individual and synergistic roles of these components may not only elucidate the mechanistic underpinnings of CFE's neuroprotective properties but could also lead to the design of more targeted and potent therapeutic interventions, leveraging the natural benefits of *Carpesii fructus*.

## 5 CONCLUSION

**FIGURE 10:**
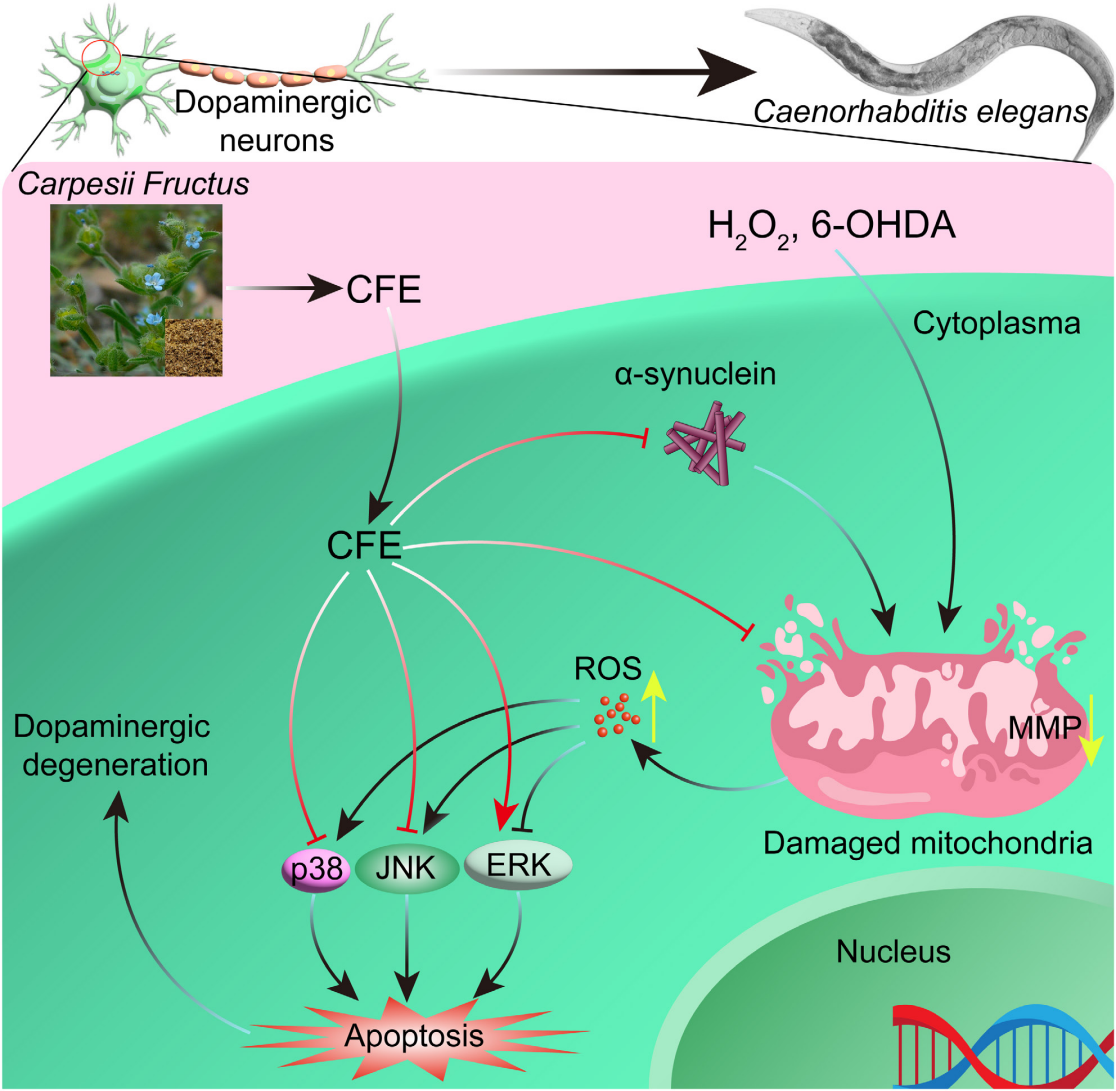
A schematic diagram of the study on CFE. CFE, a natural product, exhibits neuroprotective effects in PD models induced by H_2_O_2_ and 6‐OHDA, as well as in α‐synuclein‐expressed PC‐12 cells and *C. elegans*. This is achieved by regulating the MAPK signaling pathways, specifically by upregulating ERK and downregulating both JNK and p38 signaling pathways.

In conclusion, our current study sheds light on the potential therapeutic value of CFE as a neuroprotective agent in the context of PD via regulating the MAPK pathway (Figure 10). Furthermore, elucidation of the active compounds within CFE will offer valuable insights for the development of novel and effective therapeutic strategies targeting PD. These findings provide a strong rationale for continued research efforts to exploit CFE's neuroprotective potential and may ultimately contribute to the advancement of PD treatments in the future.

## AUTHOR CONTRIBUTIONS

Chong‐Lin Yu, Xiao‐Gang Zhou, and An‐Guo Wu conceived the ideas and designed the experiments, conceptualization, and methodology. Feng‐Dan Zhu, Bin‐Ding Wang, and Min‐Song Guo performed the experiments. An‐Guo Wu wrote the manuscript. Xiao‐Hui Su and Betty Yuen‐Kwan Law revised and edited the manuscript. Lu Yu, Da‐Lian Qin, and Jian‐Ming Wu supervised the study.

## FUNDING INFORMATION

This research was funded by grants from the National Natural Science Foundation of China (No. 81903829), the Sichuan Science and Technology Program (Nos. 2022YFS0620 and 2022YFH0115), the Macao Science and Technology Development Fund of Macao SAR (Nos. SKL‐QRCM(MUST)‐2020–2022 and MUST‐SKL‐2021‐005), and Southwest Medical University (Nos. 2021ZKZD015, 2021ZKZD018, and 2021ZKMS046).

## CONFLICT OF INTEREST STATEMENT

The authors declare that they have no competing interest.

## Supporting information


Appendix S1.

## Data Availability

The datasets used or analyzed during the current study are available from the corresponding author on reasonable request.
