# Supplementary material for: Carpesii fructus extract exhibits neuroprotective effects in cellular and Caenorhabditis elegans models of Parkinson's disease
Source: CNS Neurosci Ther. 2023 Oct 31;30(4):e14515. doi: 10.1111/cns.14515 (PMC11017466; doi:10.1111/cns.14515)
Supplement: Supplementary file 1 — Appendix S1. [file CNS-30-e14515-s001.docx]

**Supplementary materials**

***Carpesii Fructus* extract exhibits neuroprotective effects in cellular and *Caenorhabditis elegans* models of Parkinson's disease**

Feng-Dan Zhu^1,†^, Bin-Ding Wang^1,†^, Da-Lian Qin^1,†^, Xiao-Hui Su^2^, Lu Yu^1^, Jian-Ming Wu^1^, Betty Yuen-Kwan Law^3^, Min-Song Guo^1^, Chong-Lin Yu^1,*^, Xiao-Gang Zhou^1,*^ and An-Guo Wu^1,*^

*^1^ Sichuan Key Medical Laboratory of New Drug Discovery and Drugability Evaluation, Luzhou Key Laboratory of Activity Screening and Druggability Evaluation for Chinese Materia Medica, Key Laboratory of Medical Electrophysiology of Ministry of Education, School of Pharmacy, Southwest Medical University, Luzhou, China, 646000.* *lethe19970801@163.com (F.-D. Z.);* *wbinding@163.com (B.-D.W);* *dalianqin@swmu.edu.cn (D.-L.Q.); yulu863@swmu.edu.cn (L.Yu); jianmingwu@swmu.edu.cn (J.-M.W.); dididigms@163.com (M.-S.G.);* *8056ycl@swmu.edu.cn (C.-L.Y.); zxg@swmu.edu.cn (X.-G.Z.); wuanguo@swmu.edu.cn (A.-G.W.).*

*^2^Institute of Chinese Materia Medica, China Academy of Chinese Medical Sciences, Beijing, China. 100700.* *sxh66159@163.com (X.-H.S.)*

*^3^State Key Laboratory of Quality Research in Chinese Medicine, Macau University of Science and Technology, Taipa, Macau SAR, China, 99078. yklaw@must.edu.mo (B.Y.-K.L.).*

^†^Authors contribute to equal works

*Correspondence: Chong-Lin Yu, 8056ycl@swmu.edu.cn; Xiao-Gang Zhou, zxg@swmu.edu.cn; An-Guo Wu, wuanguo@swmu.edu.cn.

**
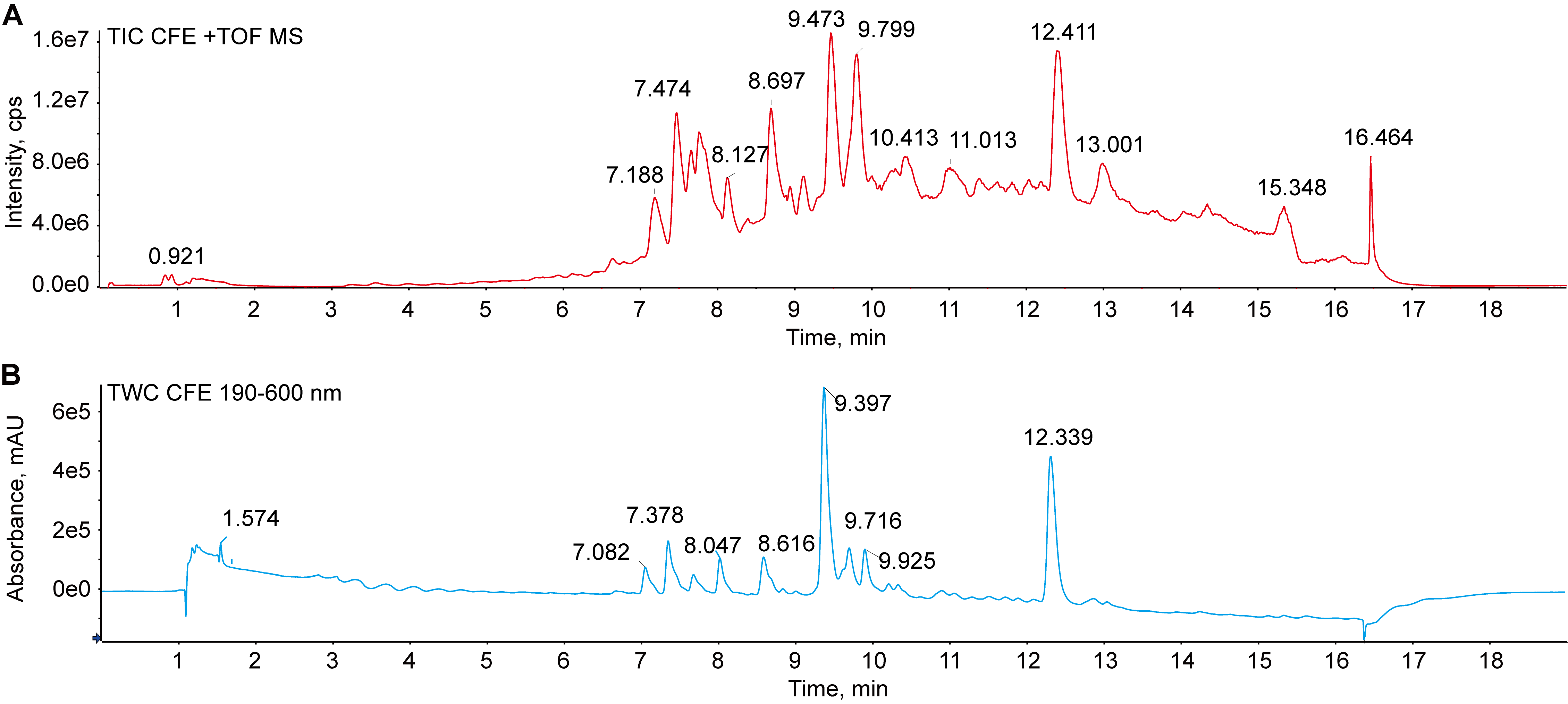
**

**Fig. S1.** UHPLC-DAD-Q/TOF-MS/MS analysis of CFE. (A) Representative total ion chromatogram (TIC) of CFE in positive ion mode. (B) Representative UHPLC-DAD chromatogram of CFE at 190-600 nm.

**
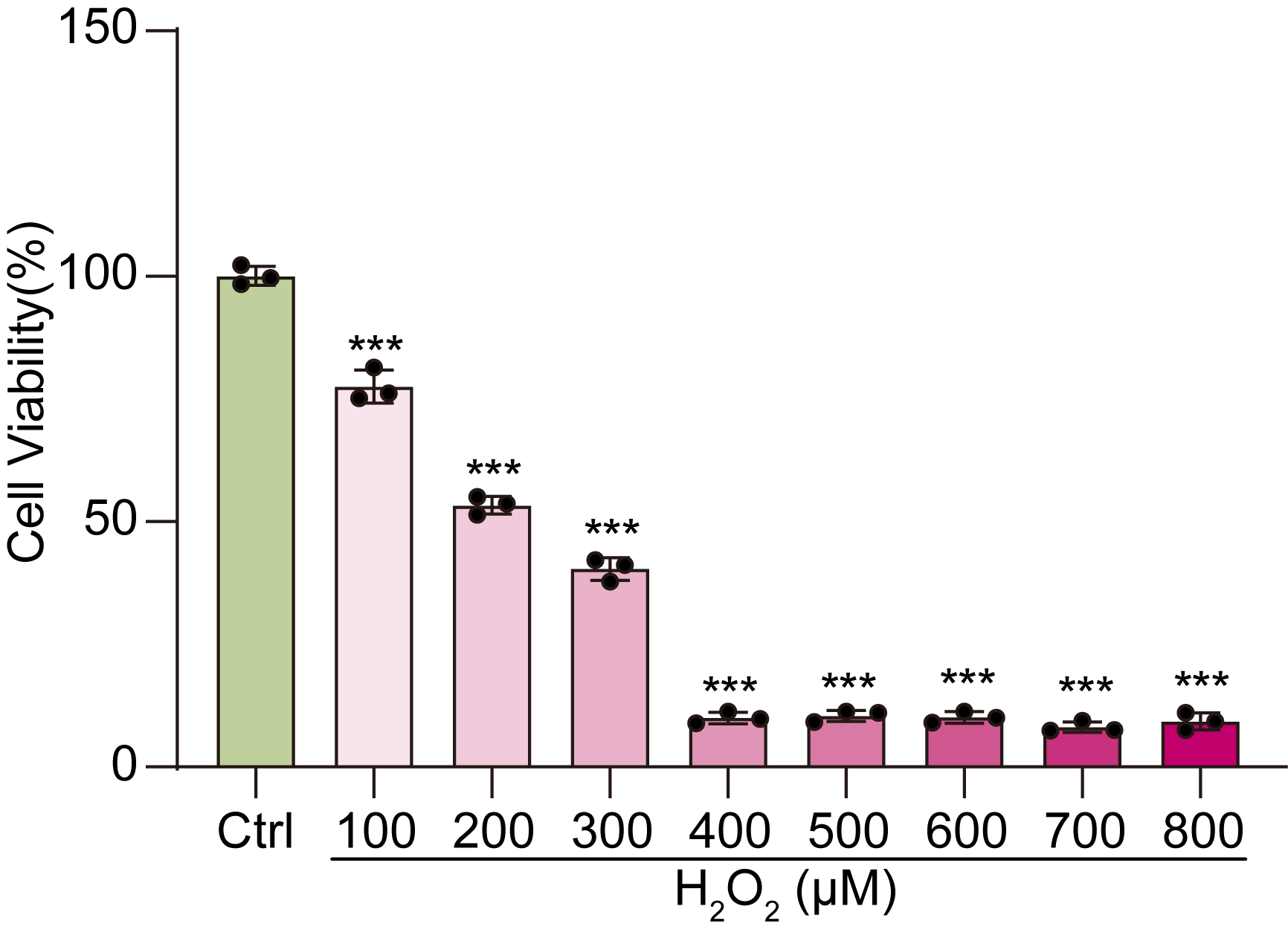
**

**Fig. S2.** The bar chart indicates the cell viability of PC-12 cells treated with H_2_O_2_ at indicated concentrations. Error bars, S.D., ****p* < 0.001.

**
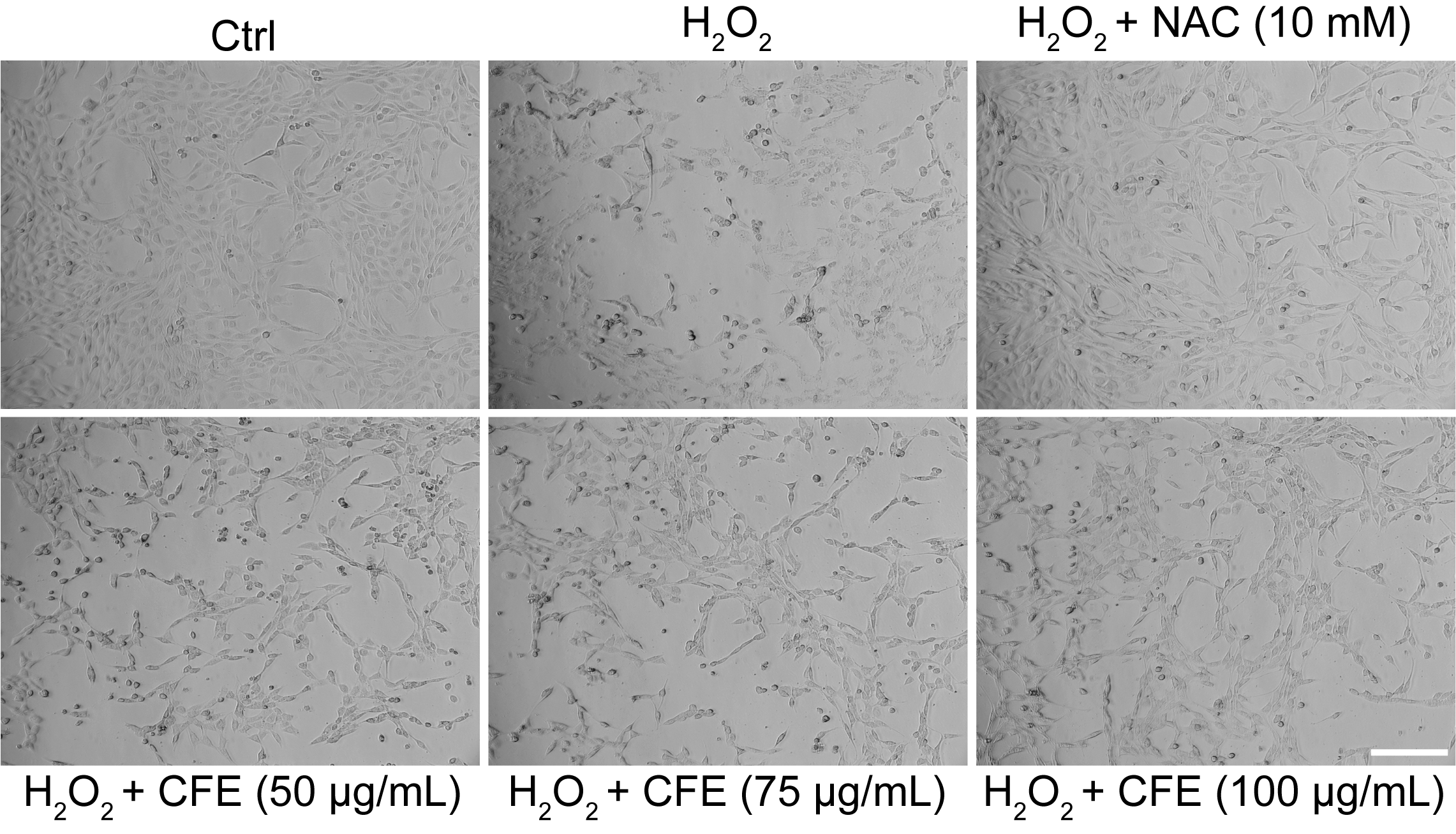
**

**Fig. S3.** Representative images of cell morphology of H_2_O_2_-treated PC-12 cells with or without CFE and NAC at indicated concentrations. Magnification: 10x, scale bar: 250 µm.

**
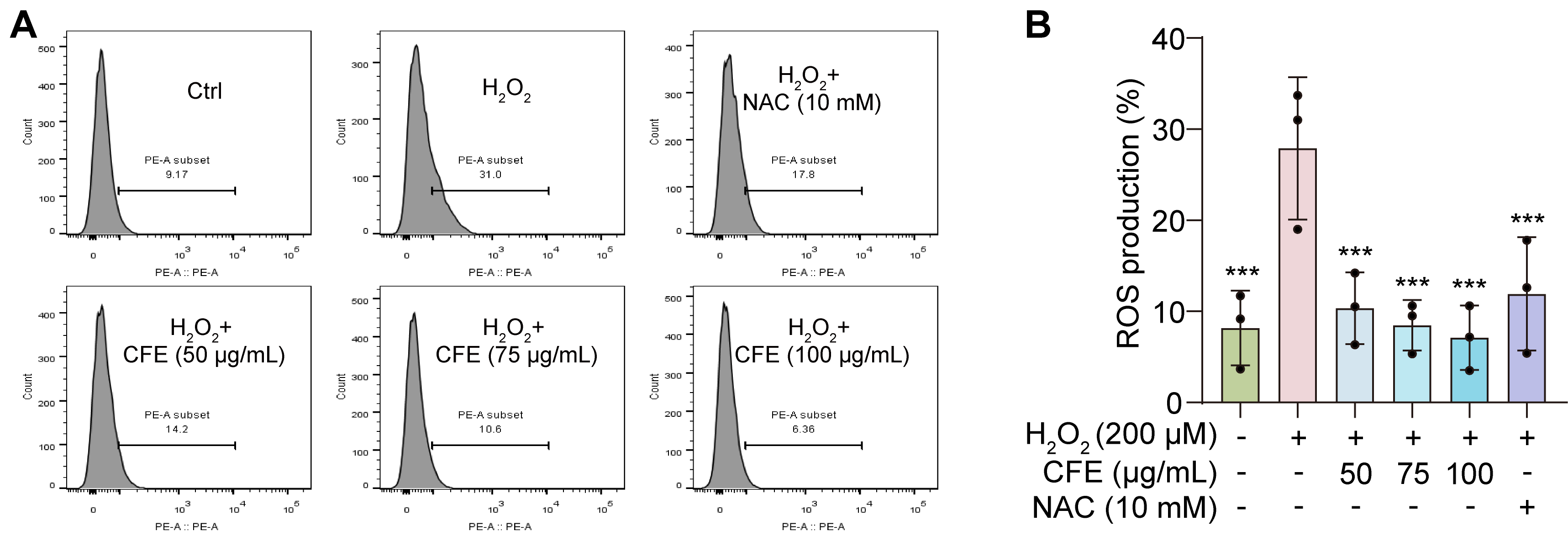
**

**Fig. S4.** Flow cytometry analysis of ROS levels in H_2_O_2_-treated PC-12 cells with or without CFE and NAC at indicated concentrations. (A) Representative images of flow cytometry analysis using DHE reagent. (B) The bar chart indicates the DHE intensity in PC-12 cells. Error bars, S.D., ****p* < 0.001.

**
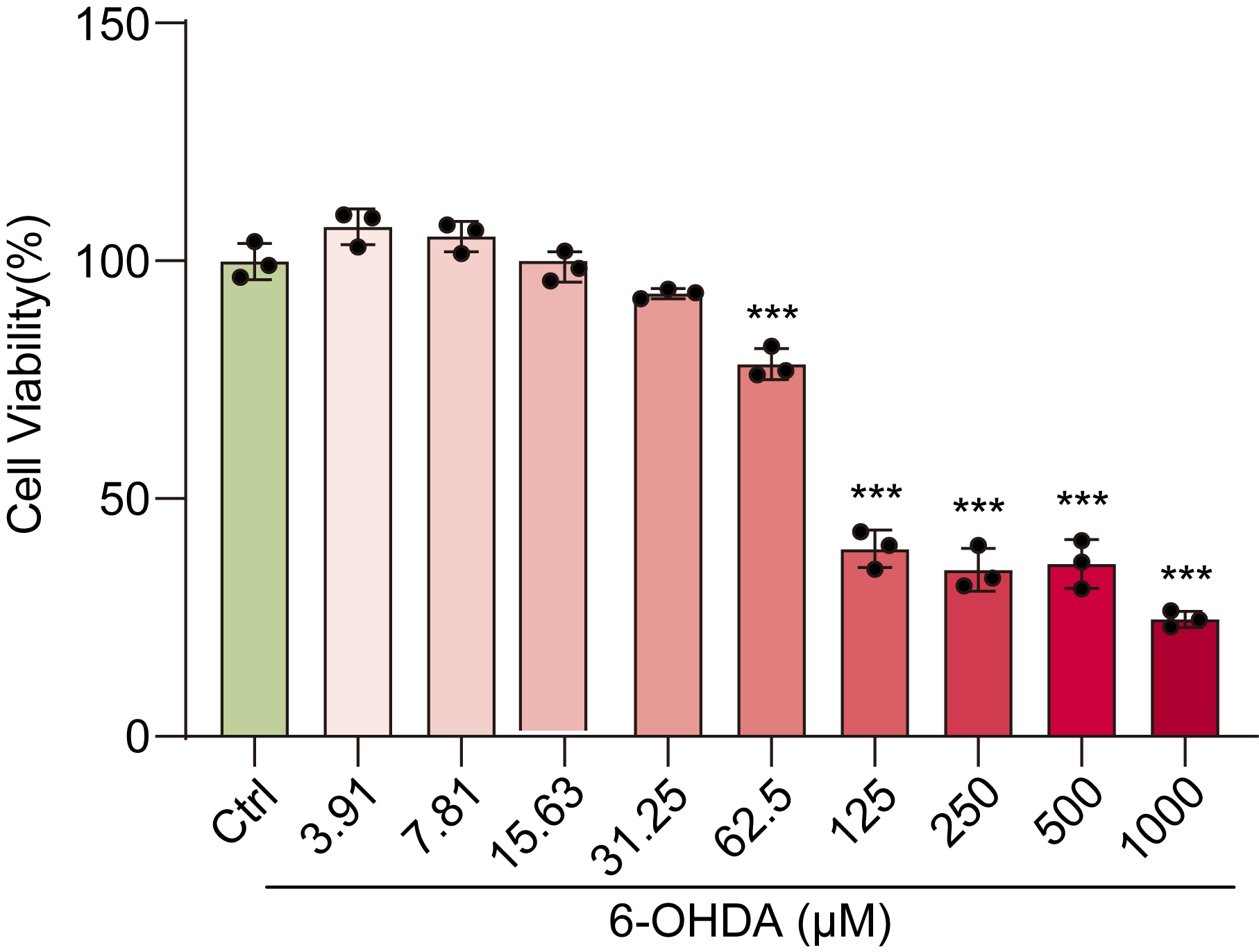
**

**Fig. S5.** The bar chart indicates the cell viability of PC-12 cells treated with 6-OHDA at indicated concentrations. Error bars, S.D., ****p* < 0.001.

**
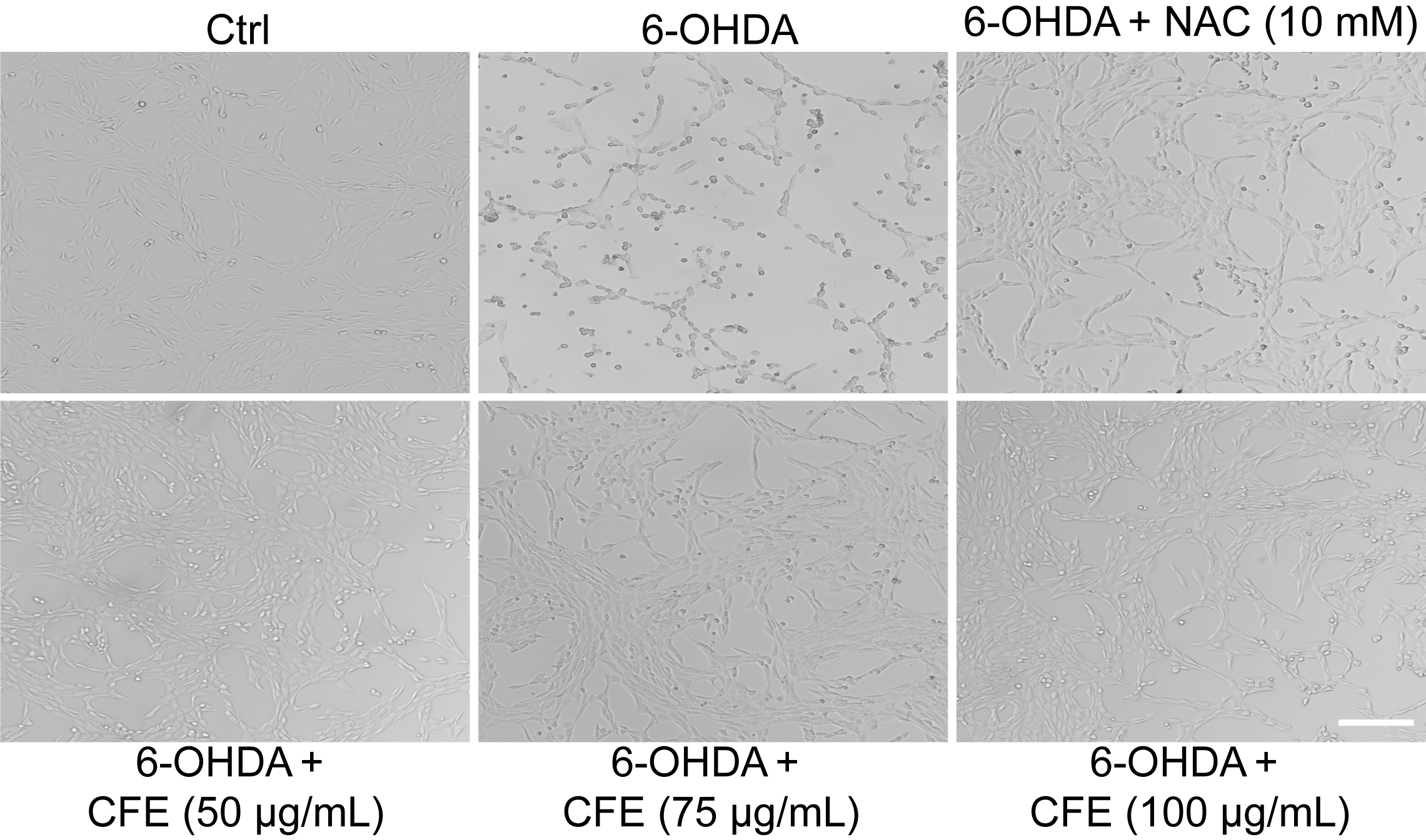
**

**Fig. S6.** Representative images of cell morphology of 6-OHDA-treated PC-12 cells with or without CFE and NAC at indicated concentrations. Magnification: 10x, scale bar: 250 µm.

**
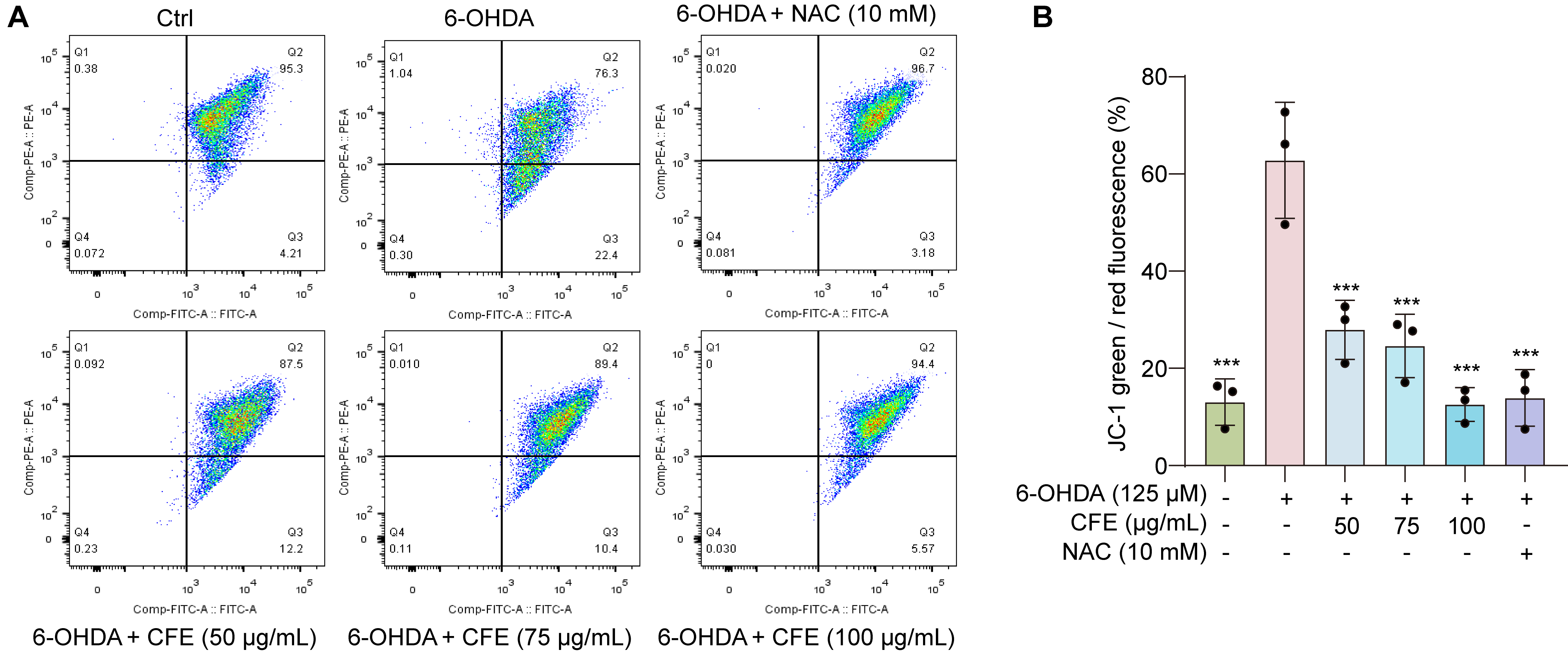
**

**Fig. S7.** Flow cytometry analysis of MMP in 6-OHDA-treated PC-12 cells with or without CFE and NAC at indicated concentrations. (A) Representative images of flow cytometry analysis using JC-1 reagent. (B) The bar chart indicates the ratio of JC-1 green to red fluorescence intensity in PC-12 cells. Error bars, S.D., ****p* < 0.001.


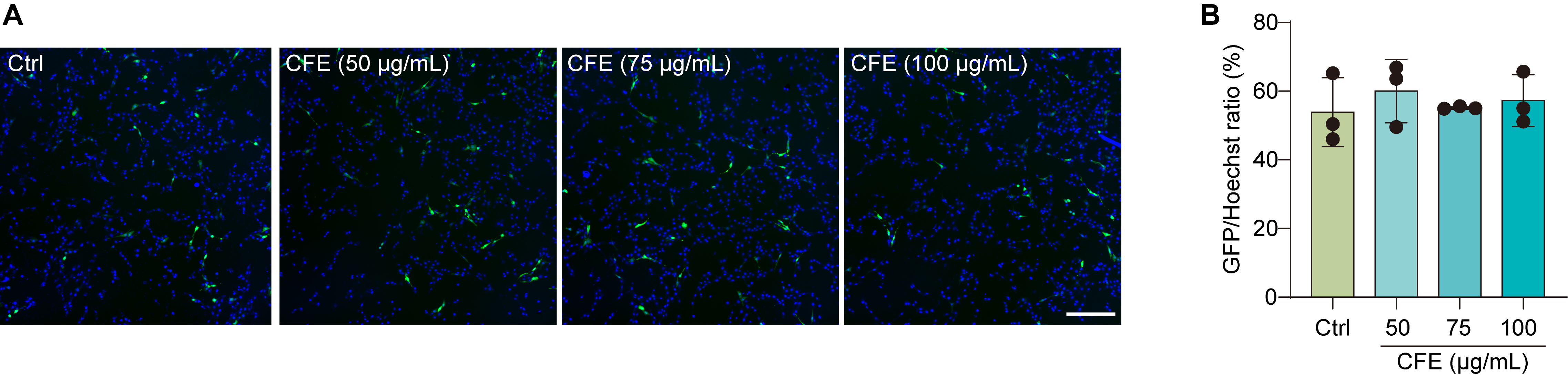


**FIGURE S8.** The effect of CFE on the transfection efficiency in EGFP-N1-transfected PC-12 cells. (A) Representative images of EGFP-N1-transfected PC-12 cells treated with or without CFE at indicated concentrations. Magnification: 10x, scale bar: 250 µm. (B) The bar chart indicates the ratio of GFP/Hoechst in PC-12 cells. Error bars, S.D.

**
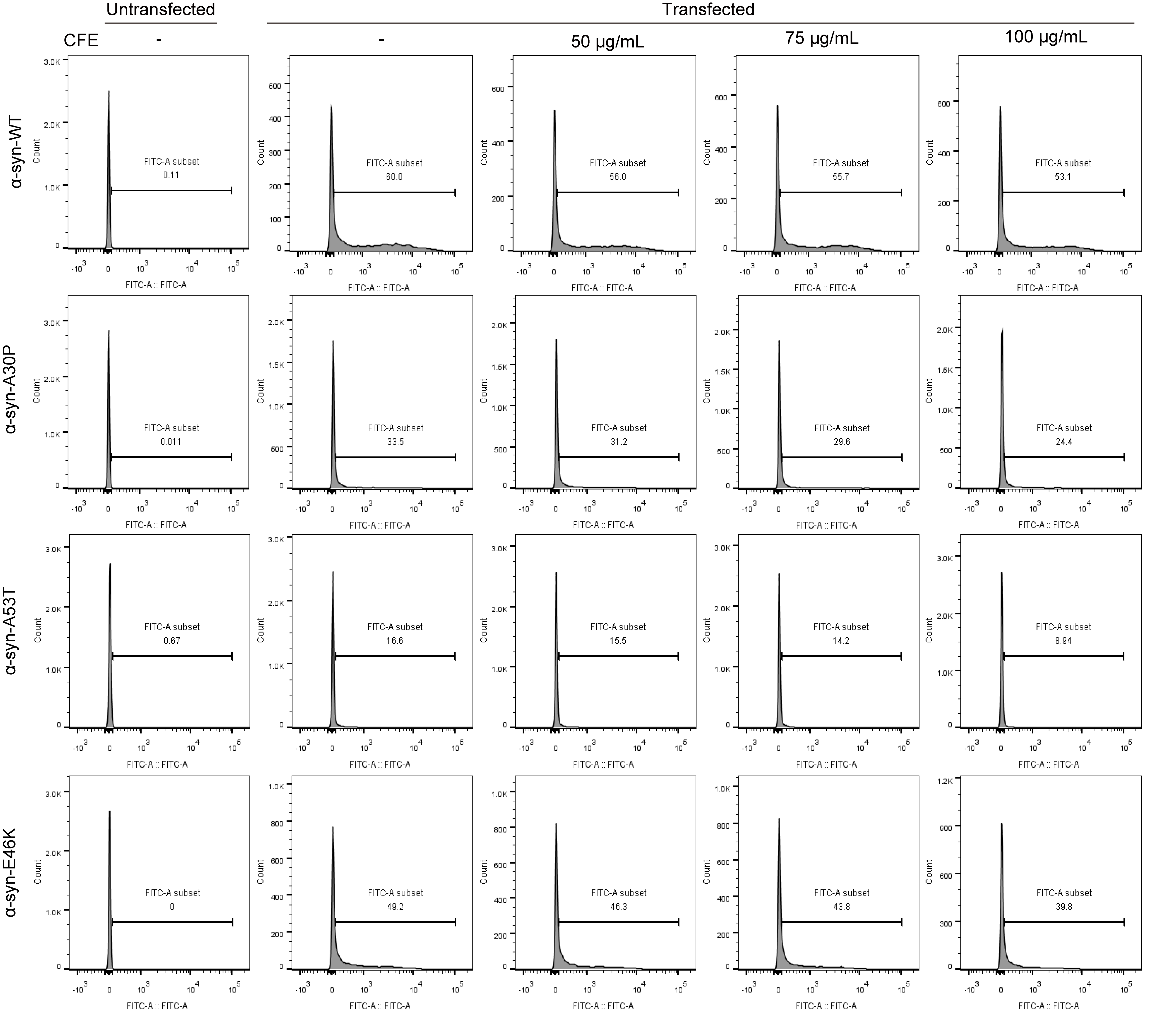
**

**Fig. S9.** Flow cytometry analysis of GFP in EGFP-α-synuclein-WT-, EGFP-α-synuclein-A30P-, EGFP-α-synuclein-A53T-, or EGFP-α-synuclein-E46K-transfected PC-12 cells. The quantifications of GFP intensity are provided in Fig. 3F-I.


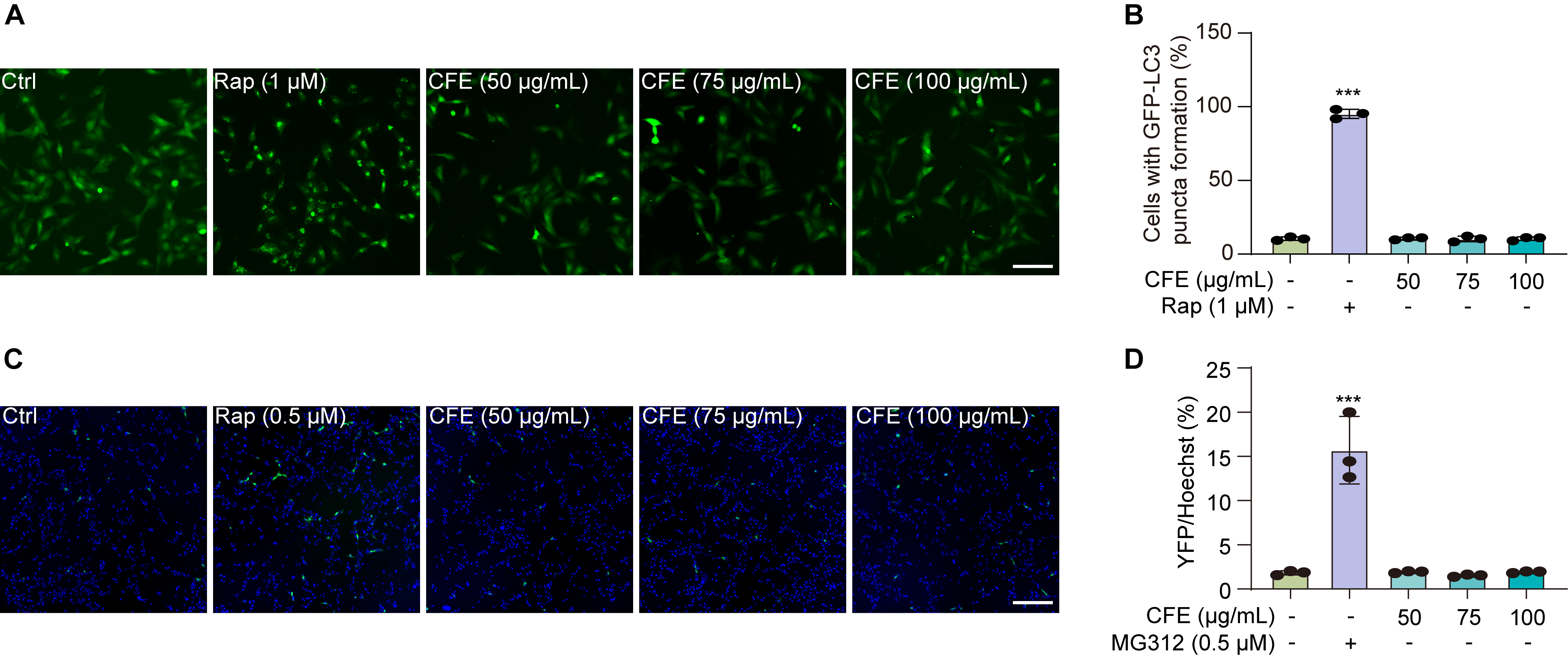


**FIGURE S10.** The effect of CFE on the activation of the autophagy-lysosome pathway (ALP) or the ubiquitin-proteasome system (UPS). (A) Representative images of GFP-LC3 stable U87 cells treated with or without rapamycin (Rap) and CFE at indicated concentrations. Magnification: 20x, scale bar: 100 µm. (B) The bar chart indicates the average number of GFP-LC3 puncta per U87 cells. (C) Representative images of YFP-CL1-transfected PC-12 cells treated with or without MG312 and CFE at indicated concentrations. Magnification: 10x, scale bar: 250 µm. (D) The bar chart indicates the ratio of YFP/Hoechst in PC-12 cells. Error bars, S.D., ****p* < 0.001.

**
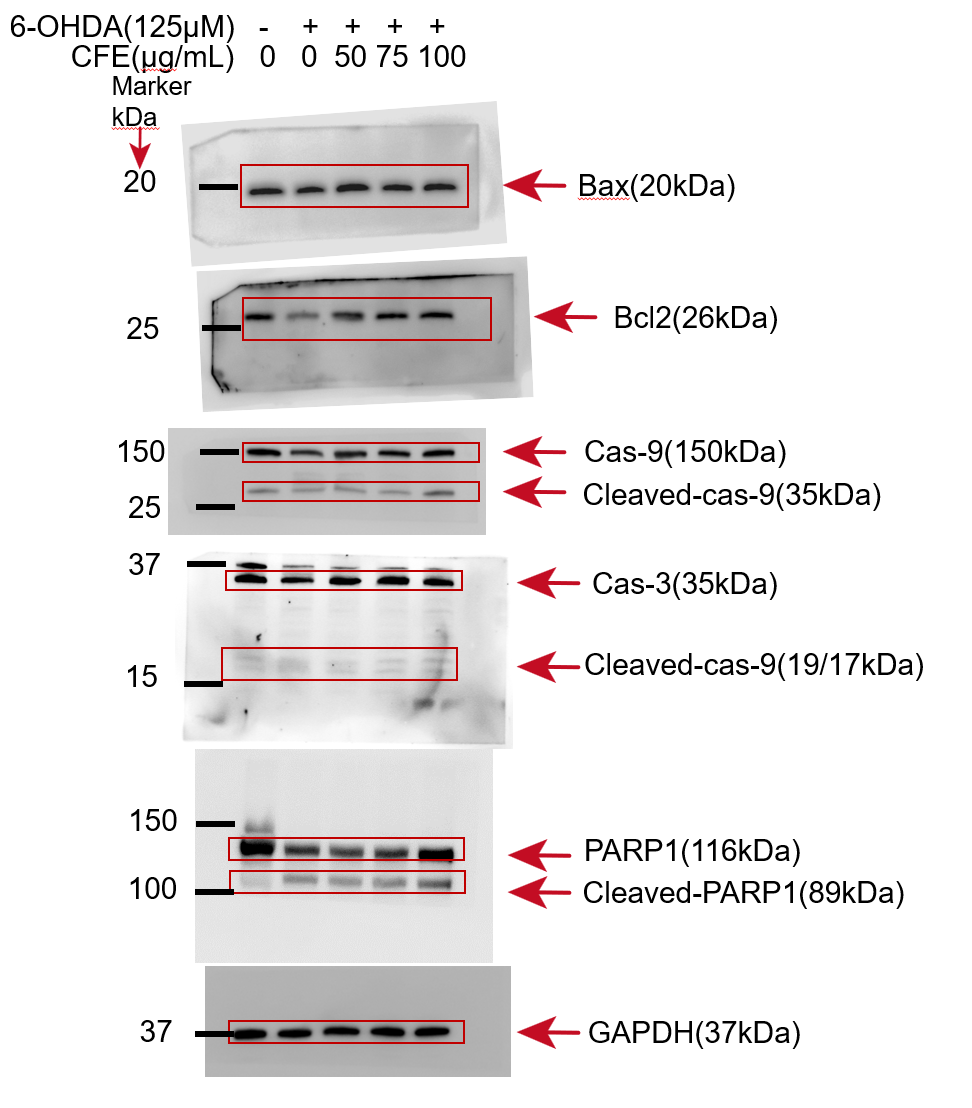
**

**Fig. S11.** Full unedited gel/blots for Figure Fig. 2I

**
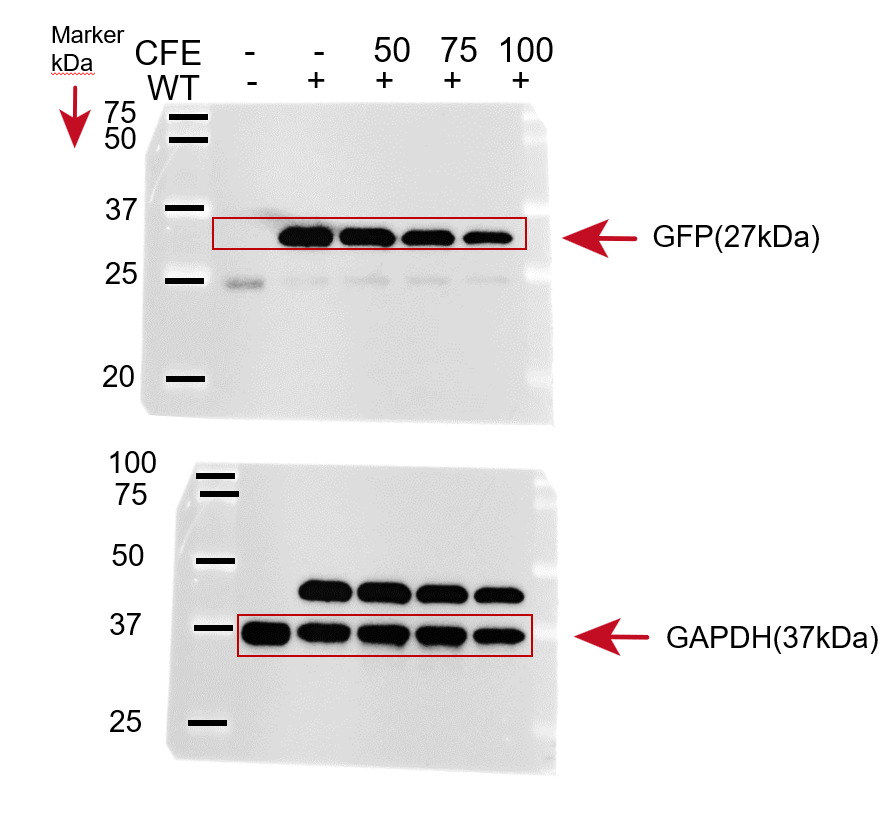
**
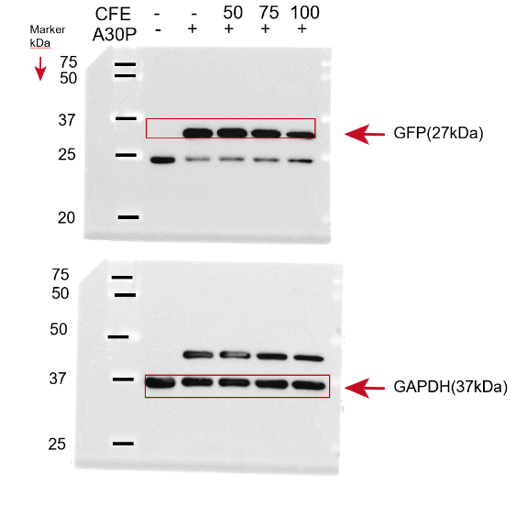


**
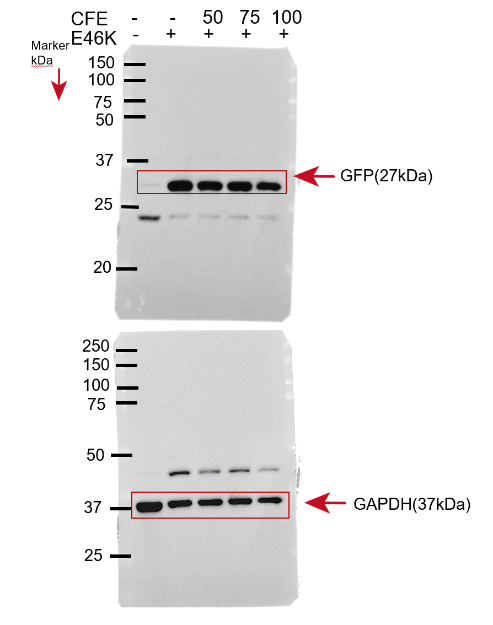

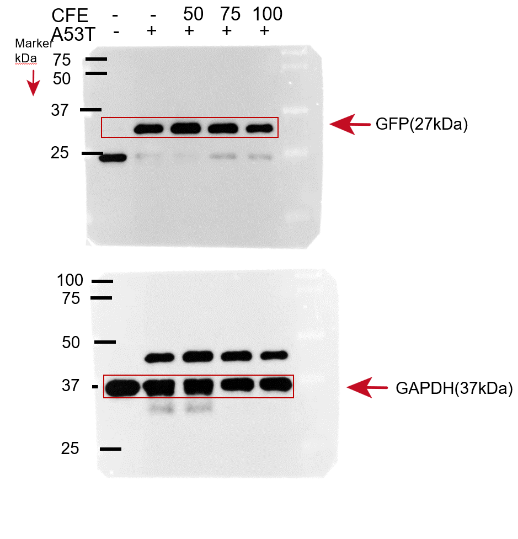
**

**Fig. S12.** Full unedited gel/blots for Figure Fig. 3J-M


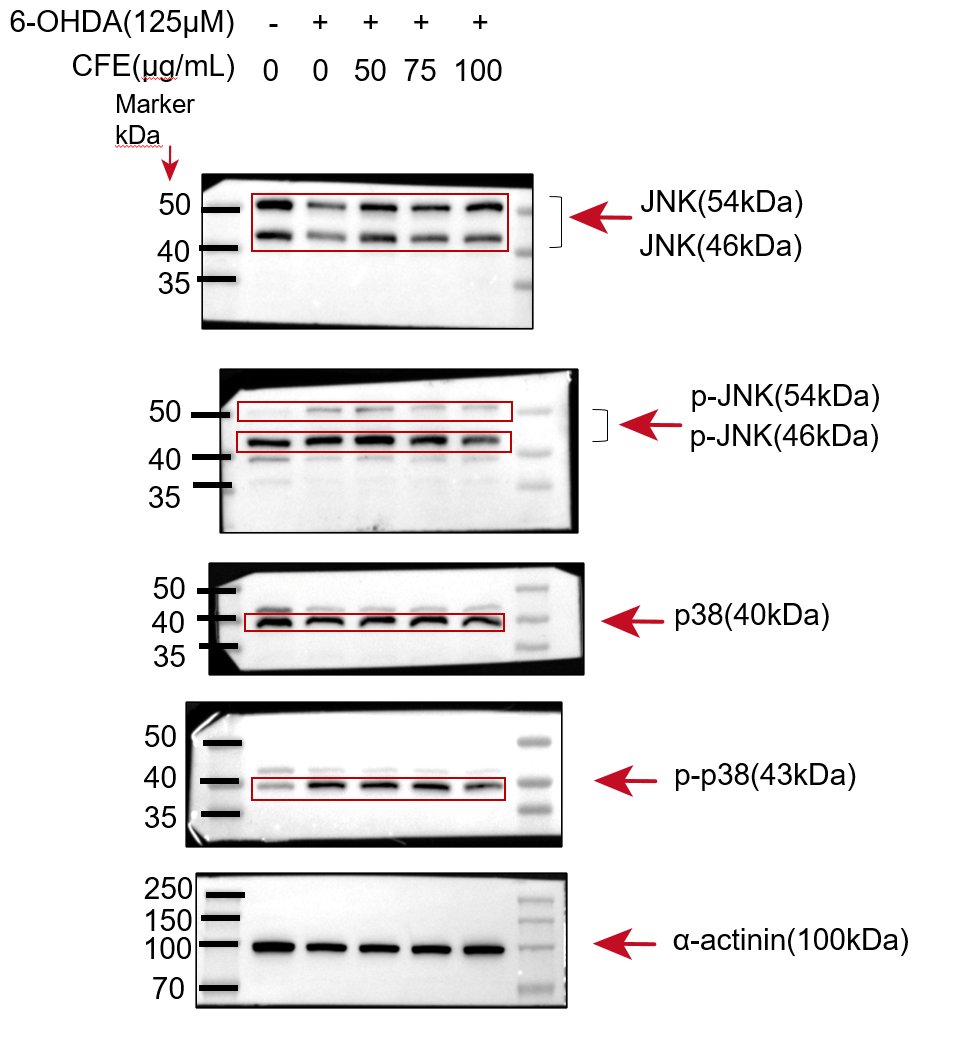
**
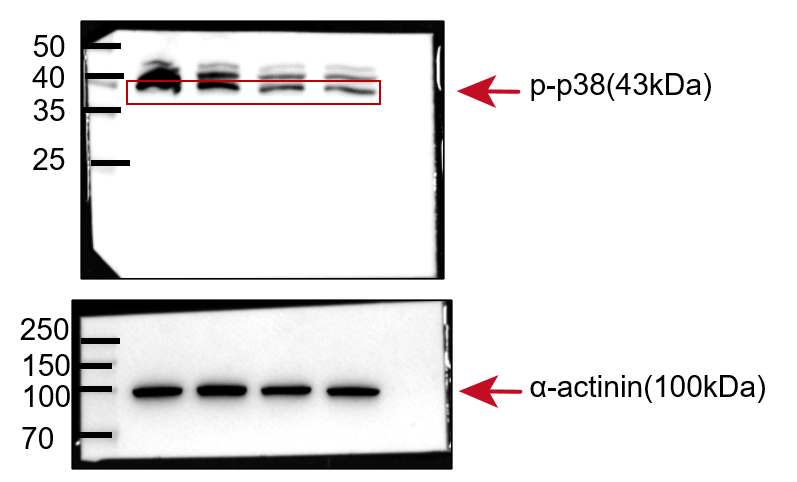

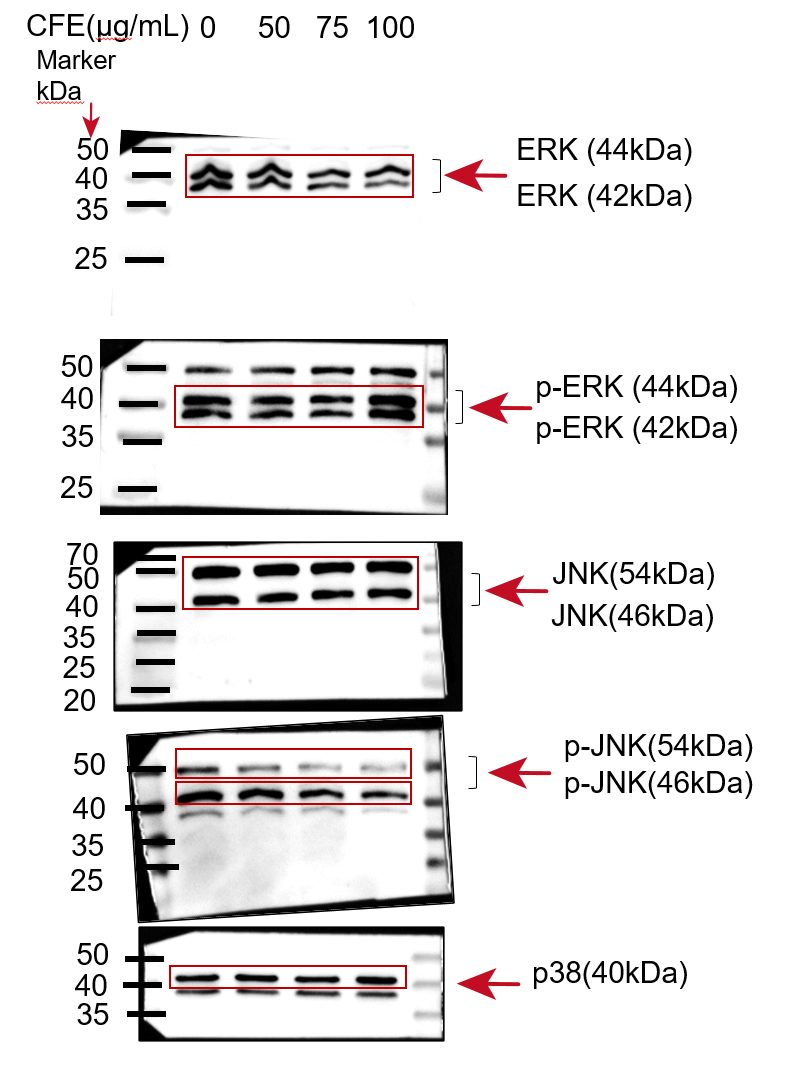
**

**Fig. S13.** Full unedited gel/blots for Figure 4A and E

**Table S1** The chemical name, retention time, chemical formula and mass of the identified potential components in CFE

| Peak No. | Retention time (min) | Chemical name | Chemical formula | Molecular weight | [M+H]^+^ |
| --- | --- | --- | --- | --- | --- |
| 1 | 0.83 | Dicarabrone A | C_30_H_40_O_6_ | 496.644 | 497.2272 |
| 2 | 0.88 | Dicarabrone B | C_30_H_40_O_6_ | 496.644 | 497.2256 |
| 3 | 0.91 | Dicarabrol | C_30_H_44_O_6_ | 500.676 | 501.1666 |
| 4 | 0.93 | Dipulchellin A | C_30_H_44_O_6_ | 500.676 | 501.1679 |
| 5 | 5.23 | Divarolide D | C_24_H_32_O_8_ | 448.512 | 449.0999 |
| 6 | 9.08 | Dicarabrol A | C_30_H_44_O7 | 516.675 | 517.1954 |
| 7 | 10.33 | 4-Hydroxy-11(13)-pseudoguaien-12,8-olide; (1α,4β,5β,8α,10α)-form, 11α,13-Dihydro, 4-O-β-D-glucopyranoside | C_21_H_34_O_8_ | 414.495 | 415.2035 |
| 8 | 11.11 | 1-Oxo-2,4(15)-eudesmadien-12,8-olide; (8β,11β)-form | C_21_H_34_O_8_ | 414.495 | 247.1287 |
| 9 | 12.29 | Ineupatorolide A | C_20_H_30_O_6_ | 366.454 | 285.0055 |
| 10 | 12.32 | Caroguaianolide C | C_15_H_20_O_5_ | 280.320 | 281.0462 |
| 11 | 12.39 | Caroguaianolide D | C_15_H_20_O_5_ | 280.320 | 281.0459 |
| 12 | 12.54 | Grandicin | C_15_H_20_O_3_ | 248.322 | 249.1442 |
| 13 | 13.65 | Carpedilactone G | C_30_H_38_O_4_ | 462.630 | 463.1225 |
| 14 | 13.66 | Caroguaianolide E | C_15_H_20_O_5_ | 280.320 | 281.046 |
| 15 | 13.71 | 5-Hydroxy-13-methoxy-4(15)-eudesmen-12,8-olide | C_16_H_24_O_4_ | 280.364 | 281.0457 |
| 16 | 13.90 | Carabrol | C_15_H_22_O_3_ | 250.338 | 251.0422 |
| 17 | 15.74 | Carabrolactone A | C_15_H_22_O_5_ | 282.336 | 283.0272 |
